# Semisynthesis and Biological Evaluation of Oleanolic Acid 3-*O*-*β*-d-Glucuronopyranoside Derivatives for Protecting H9c2 Cardiomyoblasts against H_2_O_2_-Induced Injury

**DOI:** 10.3390/molecules23010044

**Published:** 2018-01-10

**Authors:** Yu Tian, Zhonghao Sun, Wenqian Wang, Hai Shang, Baoqi Wang, Di Deng, Guoxu Ma, Haifeng Wu, Nailiang Zhu, Xudong Xu, Guibo Sun, Xiaobo Sun

**Affiliations:** 1 Beijing Key Laboratory of Innovative Drug Discovery of Traditional Chinese Medicine (Natural Medicine) and Translational Medicine, Key Laboratory of Bioactive Substances and Resources Utilization of Chinese Herbal Medicine, Ministry of Education, Key Laboratory of Efficacy Evaluation of Chinese Medicine against Glycolipid Metabolic Disorders, State Administration of Traditional Chinese Medicine, Zhong Guan Cun Open Laboratory of the Research and Development of Natural Medicine and Health Products, Institute of Medicinal Plant Development, Chinese Academy of Medical Sciences & Peking Union Medical College, Beijing 100193, China; ytian@implad.ac.cn (Y.T.); sun_zhonghao@126.com (Z.S.); hshang@implad.ac.cn (H.S.); mgxfl8785@163.com (G.M.); hfwu@implad.ac.cn (H.W.); zhu13liang@126.com (N.Z.); 2 Tianjin Institute of Pharmaceutical Research, Tianjin 300193, China; wangwq@tjipr.com; 3 Center of Research and Development on Life Sciences and Environment Sciences, Harbin University of Commerce, Harbin 150076, Heilongjiang, China; 13115539201@163.com (B.W.); 18745112039@163.com (D.D.)

**Keywords:** oleanolic acid 3-*O*-*β*-d-glucuronopyranoside, semisynthesis, derivatives, H_2_O_2_-induced, protective effect

## Abstract

A series of novel oleanolic acid 3-*O*-*β*-d-glucuronopyranoside derivatives have been designed and synthesized. Biological evaluation has indicated that some of the synthesized compounds exhibit moderate to good activity against H_2_O_2_-induced injury in rat myocardial cells (H9c2). Particularly, derivative 28-*N*-isobutyl ursolic amide 3-*O*-*β*-d-galactopyranoside (**8a**) exhibited a greater protective effect than the positive control oleanolic acid 3-*O*-*β*-d-glucuronopyranoside, indicating that it possesses a great potential for further development as a cardiovascular disease modulator by structural modification.

## 1. Introduction

Cardiovascular disease is among the leading causes of death worldwide. According to the World Health Organization, mortality from cardiovascular disease is expected to reach about 25 million by 2030 [1,2,3]. Despite the emergence of more and more listed drugs, the treatment of cardiovascular disease is still not optimistic [4,5,6,7]. Therefore, it is clinically necessary to research and develop more effective and safe agents for preventing and managing cardiovascular disease.

**Figure 1:**
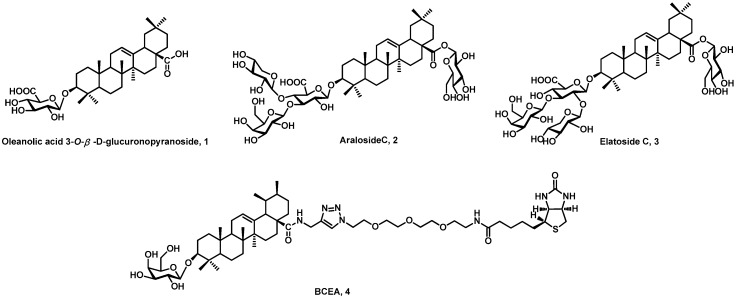
The structures of triterpenoid saponins from *Aralia elata* (Miq) Seem and the probe of oleanolic acid 3-*O*-*β*-d-glucuronopyranoside BCEA.

*Aralia elata* (Miq) Seem (AS), a kind of herbal medicine, has been used as a tonic, antiarrhythmic, anti-arthritic, antihypertensive, and anti-diabetic agent in traditional Chinese medicine [8,9]. The total saponins extracted from AS, which are found to be the main pharmacological active ingredients of AS, have been proven to exhibit anti-myocardial ischemic and anti-hypoxic activities, as well as anti-oxidative, anti-inflammatory, and anti-apoptotic capacity [10,11,12]. Moreover, as the main components of *A. elata* Xinmaitong capsules (Clinical Trial Approval Number 2003L01111 by China Food and Drug Administration), AS was developed for the treatment of coronary heart disease and has successfully completed phase III clinical trials in China [13]. Oleanolic acid 3-*O*-*β*-d-glucuronopyranoside (**1**, Figure 1) is one of the major natural pentacyclic triterpenoid saponins isolated from AS [14,15]. We previously demonstrated that the triterpenoid saponins araloside C (**2**, Figure 1) and elatoside C (**3**, Figure 1) from AS can protect myocardial cells from ischemia/reperfusion (IR) injury and reduce I/R-induced oxidative stress and apoptosis in cardiomyoblasts [16,17,18]. We also reported that the proteomic profiling of oleanolic acid 3-*O*-*β*-d-glucuronopyranoside targets is associated with anti-apoptotic effects in endothelial cells by using biotinconjugated calenduloside E analogue (BCEA, **4**, Figure 1) [19]. However, there are no reports about the protective effects of oleanolic acid 3-*O*-*β*-d-glucuronopyranoside synthetic derivatives on cardiomyoblast damage.

**Figure 2:**
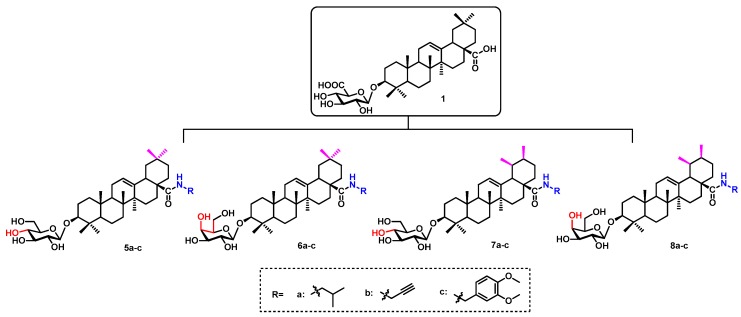
The structures of oleanolic acid 3-*O*-*β*-d-glucuronopyranoside derivatives.

In this current study, we describe the preparation of a series of novel oleanolic acid 3-*O*-*β*-d-glucuronopyranoside derivatives with several amine moieties (isobutylamine, propynylamine, and 3,4-dimethoxybenzylamine) that are appended to the C-28 carboxyl group of the parental compound. Meanwhile, in order to extend the activity scaffold of oleanolic acid 3-*O*-*β*-d-glucuronopyranoside and improve its cardiovascular protective effects, we planned to change the aglycone moiety from oleanane to ursane, which is another natural pentacyclic triterpenoid that possesses biological activity. To further optimize the potency of oleanolic acid 3-*O*-*β*-d-glucuronopyranoside derivatives and to gain further insight into their structure–activity relationship, we also designed the analogues that derive from glycosylation at the C-3 position with d-glucose and d-galactose. For this purpose, herein we synthesized a series of novel oleanolic acid 3-*O*-*β*-d-glucuronopyranoside amide derivatives (**5a**–**c**, **6a**–**c**, **7a**–**c**, **8a**–**c**, Figure 2) and evaluated their protective activities as cardiovascular disease agents.

## 2. Results and Discussion

### 2.1. Chemistry

**Scheme 1:**
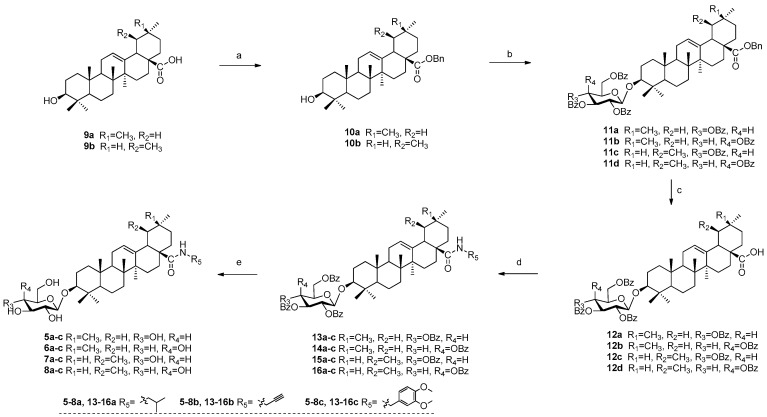
The synthesis of target compounds. Reagents and conditions: (a) BnBr, K_2_CO_3_, TBAB, DCM-H_2_O, r.t., 18 h; (b) glycosyl donors, TMSOTf, 4 Å MS, DCM, r.t., 2–4 h; (c) H_2_, Pd–C (10%), EtOAc, reflux, 4–6 h; (d) HOBt, EDCI, R_5_NH_2_, r.t., 4–16 h; (e) NaOMe, MeOH, r.t., 2–3 h.

The synthesis of derivatives **5a**–**c**, **6a**–**c**, **7a**–**c** and **8a**–**c** is outlined in Scheme 1. The naturally abundant oleanolic acid (**9a**) and ursolic acid (**9b**) were treated with benzyl bromide (BnBr), potassium carbonate solution (K_2_CO_3_), and tetrabutylammonium bromide (TBAB) in dry dichloromethane (DCM) to obtain **10a** and **10b**, respectively. Compounds **10a** and **10b** reacted with glucosyl donors and galactosyl donors in Lewis acid trimethylsilyl trifluoromethanesulfonate (TMSOTf) conditions to provide compounds **11a**–**d**, which were subjected to several hydrogenations to obtain compounds **12a**–**d** in the presence of catalytic amounts of 10% Pd–C at atmospheric pressure. Compounds **13a**–**c**, **14a**–**c**, **15a**–**c** and **16a**–**c** were attained via amidation with various amines of the C-28 carboxyl group of the saponin scaffold, and then followed by deprotection of the glycosyl groups in the presence of NaOMe/MeOH solution to gain compounds **5a**–**c**, **6a**–**c**, **7a**–**c** and **8a**–**c**. The above reaction conditions have also been described in our previous paper [19].

### 2.2. Biological Results and Discussion

**Figure 3:**
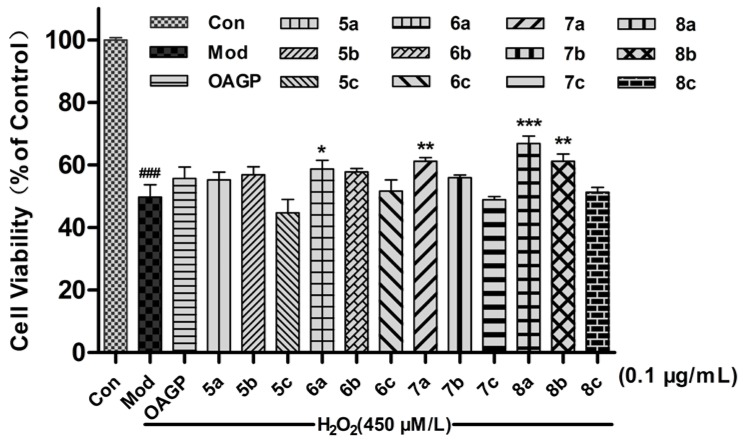
Effects of oleanolic acid 3-*O*-*β*-d-glucuronopyranoside derivatives on H_2_O_2_-induced cell damage in H9c2 cells. H9c2 cardiomyoblasts were pre-incubated with a 0.1 μg/mL concentration of derivatives (**5a**–**c**, **6a**–**c**, **7a**–**c** and **8a**–**c**) for 12 h and then exposed to H_2_O_2_ (450 μmol/L) for 1 h. The data are expressed as the mean ± S.D. from three independent experiments. ^### ^*p* < 0.001 versus control group; * *p* < 0.05 versus H_2_O_2_-treated cells; ** *p* < 0.01 versus H_2_O_2_-treated cells; *** *p* < 0.001 versus H_2_O_2_-treated cells.

The protective effect of oleanolic acid 3-*O*-*β*-d-glucuronopyranoside and its derivatives against H_2_O_2_-induced cell injury was detected using the 3-(4,5-dimethylthiazol-2-yl)-2,5-diphenyltetrazolium bromide (MTT) assay. MTT results show that some compounds exhibited moderate to good protective effects against H_2_O_2_-induced injury in H9c2 cells. As shown in Figure 3, the preliminary test of compounds **5a**–**c**, **6a**–**c**, **7a**–**c** and **8a**–**c** at 0.1 μg/mL revealed that **5a**–**b**, **6a**–**b**, **7a**–**b** and **8a**–**b** better increased the viability of H9c2 cells. Pretreatment of H9c2 cells with compounds **6**–**8a** and **8b** exhibited a better protective effect than oleanolic acid 3-*O*-*β*-d-glucuronopyranoside (OAGP). The survival rate increased from 49.69% (with H_2_O_2_ treatment alone) to 58.69%, 61.21%, 66.86% and 61.19% after pretreatment with 0.1 μg/mL compounds** 6**–**8a** and **8b**, respectively. Among them, analogue **8a** exhibited a more potent cytoprotective effect than the others after pretreatment for 12 h, which suggests that **8a** deserves further evaluation as a potential therapeutic agent for protection against H_2_O_2_-induced injury in H9c2 cells.

The preliminary structure activity relationships (SARs) suggest that amide derivatives of oleanolic acid 3-*O*-*β*-d-glucuronopyranoside containing ursane scaffolds were more potent than those containing oleanane scaffolds (e.g., compounds **7**–**8a** vs. **5**–**6a**). Beyond that, the compounds **5a**–**b**, **6a**–**b**, **7a**–**b** and **8a**–**b** with isobutylamine and propynylamine groups could increase cell viability after H_2_O_2_ treatment, compared to the control, indicating that the introduction of these aliphatic substituent groups could increase cell viability. However, compounds **5a**–**c**, including the 3,4-dimethoxybenzylamine group, were inert, suggesting that the unsaturated aryl groups were the adverse substituent group in the derivatives. In addition, these above amide analogs possessed protective potency, indicating that the C-28 carboxyl group was not an essential group for protective activity, and the substituents on the amide nitrogen affected the cell viability obviously.

## 3. Experimental Section

### 3.1. General Information

All the reagents were used without further purification unless otherwise specified. Solvents were dried and redistilled in the usual manner prior to use. Analytical TLC was performed using silica gel HF254. Preparative column chromatography was performed with silica gel H. ^1^H- and ^13^C-NMR spectra were recorded on a Bruker Advance III 600 MHz spectrometer. HRMS were obtained on a Thermo Fisher LTQ-Obitrap XL. Oleanolic acid 3-*O*-*β*-d-glucuronopyranoside was provided by the Institute of Medicinal Plant Development (Beijing, China) [20]. Cell culture products were purchased from Gibco BRL (Grand Island, NY, USA).

### 3.2. Chemistry

The synthesis of the glucosyl donor and galactosyl donor was described [20].

#### 3.2.1. General Procedure for the Synthesis of Compounds **10a**–**10b**

To a solution of oleanolic acid (**9a**) or ursolic acid (**9b**) (10.0 g, 21.8 mmol) in dry DCM (300 mL), TBAB (0.8 g, 2.5 mmol) and K_2_CO_3_ (7.4 g, 53.6 mmol) in water (50 mL) were added, and benzyl bromide (3.2 mL, 26.8 mmol) was dropped at 0 °C. Then, the reaction mixture was stirred at room temperature for 18 h. The reaction was monitored by TLC. The crude mixture was separated and the water layer was extracted with DCM (3 × 100 mL). The combined organic layer was washed with 0.1 mol/L HCl aqueous solution, NaHCO_3_ saturated aqueous solution, and NaCl saturated aqueous solution in sequence, and then dried over Na_2_SO_4_ and purified through column chromatography (eluent: PE-EtOAc, 8:1) to offer pure white solids **10a** (10.8 g, 91% yield) and **10b** (11.1 mg, 93% yield).

#### 3.2.2. General Procedure for the Synthesis of Compounds **11a**–**11d**

To a solution of compound **10a** or **10b** (3.3 g, 6.0 mmol) in dry DCM (50 mL), glucosyl donor or galactosyl donor (5.8 g, 7.9 mmol) and 4 Å molecular sieve 0.5 g were added and stirred at room temperature for 1 h under N_2_ air. Then, Lewis acid TMSOTf (60 μg, 0.3 mmol) was dropped and reacted for 2–4 h. When complete, 1.0 mL triethylamine was added to quench the reaction. Then, the suspension was filtered and the filtrate was evaporated and the crude product was subjected to column chromatography (eluent: PE-EtOAc, 10:1) to gain the pure compounds **11a** (5.3 g, 79% yield), **11b** (5.1 g, 75% yield), **11c** (4.7 g, 69% yield), and **11d** (4.7 g, 70% yield) as white solids.

#### 3.2.3. General Procedure for the Synthesis of Compounds **12a**–**12d**

A mixture of **11a**–**11d** (3.0 g, 2.6 mmol) and 10% Pd/C (1.5 mg) was hydrogenated at 1 atm for 4–6 h in refluxing EtOAc (30 mL). The mixture was filtered and concentrated and the residue was purified by silica gel column chromatography (eluent: PE-EtOAc, 3:1) to get the pure compounds **12a** (2.5 g, 92% yield), **12b** (2.5 g, 93% yield), **12c** (2.4 g, 90% yield), and **12d** (2.4 g, 91% yield) as white solids.

#### 3.2.4. General Procedure for the Synthesis of Compounds **13a**–**c**, **14a**–**c**, **15a**–**c**, **16a**–**c** and compounds **5a**–**c**, **6a**–**c**, **7a**–**c**, **8a**–**c**

To a solution of compounds **12a**–**12d** (1.0 g, 0.98 mmol) in dry DCM (15 mL), HOBt (0.2 g, 1.46 mmol) and EDCI (0.28 g, 1.46 mmol) were added and stirred at room temperature for 1 h. To this mixture, various amines (3.92 mmol) were added respectively at 0 °C and the reaction mixture was stirred 4–16 h until its completion. The solvent was washed with 0.1 mol/L HCl aqueous solution, NaHCO_3_ saturated aqueous solution, and NaCl saturated aqueous solution in sequence, and then dried over Na_2_SO_4_. The suspension was filtered and the filtrate was concentrated to give compounds **13a–c**, **14a–c**, **15a–c** and **16a–c**, which were used without further purification. To a solution of compounds **13a–c**, **14a–c**, **15a–c** and **16a–c** in MeOH/DCM (8 mL, 3:1) was added 1 mol/L NaOMe/NaOH solvent (1.6 mL). The reaction mixture was stirred for 2–3 h until its completion, after that Amberlite IR-120 was added to acidate pH 7. The suspension was filtered and the filtrate was evaporated and purified through column chromatography (eluent: DCM-CH_3_OH, 10:1) to offer pure white solids **5a–c**, **5a–c**, **7a–c**, and **8a–c**.

*28-N-Isobutyl oleanolic amide 3-O-β-d-glucopyranoside *(**5a**): White solid, 88% yield; [α]${}_{D}^{25}$: +56.25 (c 0.13, MeOH); ^1^H-NMR (600 MHz, pyridine-*d*_5_) *δ*: 7.25 (t, *J* = 5.8 Hz, 1H, N-H), 5.47 (t, *J* = 3.3 Hz, 1H, H-12), 4.96 (d, *J* = 7.8 Hz, 1H, H-1′), 4.61 (dd, *J* = 11.6 Hz, 2.1 Hz, 1H, Glu-H), 4.44 (dd, *J* = 11.6 Hz, 5.2 Hz, 1H, Glu-H), 4.29–4.23 (m, 2H, Glu-H), 4.08–4.03 (m, 2H, Glu-H), 3.46–3.41 (m, 2H, H-3, H-31-1), 3.12–3.07 (m, 2H, H-18, H-31-2), 1.35 (s, 3H, CH_3_), 1.30 (s, 3H, CH_3_), 1.04 (s, 3H, CH_3_), 0.97 (s, 6H, 2 × CH_3_), 0.95 (s, 3H, CH_3_), 0.91 (d, *J* = 6.9 Hz, 6H, H-33, H-34), 0.90 (s, 3H, CH_3_); ^13^C-NMR (150 MHz, pyridine-*d*_5_) *δ*: 177.5, 145.1, 122.8, 107.0, 88.9, 78.8, 78.4, 75.9, 71.9, 63.1, 55.8, 48.0, 47.3, 46.8, 46.6, 42.3, 42.1, 39.8, 39.6, 38.8, 37.0, 34.5, 33.9, 33.2 (C × 2), 30.9, 28.8, 28.3, 28.0, 26.6, 26.2, 23.9, 23.8 (C × 2), 20.5 (C × 2), 18.5, 17.5, 17.1, 15.5; HRMS (ESI): Calcd for [M + Na]^+^ C_40_H_67_NNaO_7_: 696.4815, found 696.4811.

*28-N-Propargyl oleanolic amide 3-O-β-d-glucopyranoside* (**5b**): White solid, 75% yield; [α]${}_{D}^{25}$: +23.25 (c 0.13, MeOH); ^1^H-NMR (600 MHz, pyridine-*d*_5_) *δ*: 8.03 (t, *J* = 5.5 Hz, 1H, N-H), 5.43 (t, *J* = 3.5 Hz, 1H, H-12), 4.96 (d, *J* = 7.7 Hz, 1H, H-1′), 4.60 (dd, *J* = 11.6 Hz, 2.3 Hz, 1H, Glu-H), 4.44–4.38 (m, 2H, H-31-1, Glu-H), 4.33–4.23 (m, 3H, H-31-2, Glu-H), 4.07–4.01 (m, 2H, Glu-H), 3.41 (dd, *J* = 11.8 Hz, 4.4 Hz, 1H, H-3), 3.14 (dd, *J* = 13.6 Hz, 4.4 Hz, 1H, H-18), 3.09 (t, *J* = 2.5 Hz, 1H, H-33), 1.34 (s, 3H, CH_3_), 1.29 (s, 3H, CH_3_), 1.03 (s, 3H, CH_3_), 1.01 (s, 3H, CH_3_), 0.93(s, 6H, 2 × CH_3_), 0.88 (s, 3H, CH_3_); ^13^C-NMR (150 MHz, pyridine-*d*_5_) *δ*: 177.3, 144.7, 122.9, 106.8, 88.8, 82.1, 78.7, 78.2, 75.7, 71.8, 71.7, 63.0, 55.7, 47.9, 46.7, 46.4, 42.1, 41.7, 39.8, 39.4, 38.7, 36.9, 34.3, 33.5, 33.1, 33.0, 30.8, 29.1, 28.2, 27.8, 26.5, 26.1, 23.7, 23.6 (C × 2), 18.4, 17.6, 17.0, 15.4; HRMS (ESI): Calcd for [M + H]^+ ^C_39_H_62_NO_7_: 656.4526, found 656.4514.

*28-N-(3*′*,4*′*-Dimethoxybenzyl) oleanolic amide 3-O-β-d-glucopyranoside *(**5c**): White solid, 77% yield; [α]${}_{D}^{25}$: +21.00 (c 0.13, MeOH); ^1^H-NMR (600 MHz, pyridine-*d*_5_) *δ*: 7.90 (t, *J* = 5.7 Hz, 1H, N-H), 7.12 (d, *J* = 1.8 Hz, 1H, H-2′′), 7.06 (dd, *J* = 8.1 Hz, 1.8 Hz, 1H, H-6′′), 6.92 (d, *J* = 8.2 Hz, 1H, H-5′′), 5.46 (t, *J* = 3.3 Hz, 1H, H-12), 4.97 (d, *J* = 7.8 Hz, 1H, H-1′), 4.79 (dd, *J* = 14.5 Hz, 5.9 Hz, 1H, H-31-1), 4.62–4.56 (m, 2H, H-31-2, Glu-H), 4.44 (dd, *J* = 11.6 Hz, 5.4 Hz, 1H, Glu-H), 4.30–4.24 (m, 2H, Glu-H), 4.08–4.02 (m, 2H, Glu-H), 3.77 (s, 3H, Ph-OMe), 3.74 (s, 3H, Ph-OMe), 3.42 (dd, *J* = 11.8 Hz, 4.3 Hz, 1H, H-3), 3.18 (dd, *J* = 13.1 Hz, 4.0 Hz, 1H, H-18), 1.35 (s, 3H, CH_3_), 1.31 (s, 3H, CH_3_), 1.03 (s, 3H, CH_3_), 0.95 (s, 6H, 2 × CH_3_), 0.91 (s, 3H, CH_3_), 0.89 (s, 3H, CH_3_); ^13^C-NMR (150 MHz, pyridine-*d*_5_) *δ*: 177.4, 149.1, 144.9, 133.3, 122.8, 120.5, 112.7, 106.8, 88.8, 78.7, 78.2, 75.8, 71.9, 63.1, 56.0, 55.8 (C × 2), 47.9, 46.8, 46.5, 43.4, 42.2, 42.0, 39.8, 39.5, 38.7, 37.0, 34.4, 33.8, 33.1, 33.0, 30.8, 28.2, 27.9, 26.5, 26.1, 23.8 (C × 2), 23.7, 18.4, 17.4, 17.0, 15.4; HRMS (ESI): Calcd for [M + Na]^+ ^C_45_H_69_NNaO_9_: 790.4870, found 790.4861.

*28-N-Isobutyl oleanolic amide 3-O-β-d-galactopyranoside *(**6a**): White solid, 87% yield; [α]${}_{D}^{25}$: +63.00 (c 0.13, MeOH); ^1^H-NMR (600 MHz, pyridine-*d*_5_) *δ*: 7.26 (t, *J* = 5.8 Hz, 1H, N-H), 5.47 (t, *J* = 3.3 Hz, 1H, H-12), 4.88 (d, *J* = 7.8 Hz, 1H, H-1′), 4.61–4.60 (m, 1H, Gal-H), 4.52–4.46 (3H, m, Gal-H), 4.19 (dd, *J* = 3.4 Hz, 9.5 Hz, 1H, Gal-H), 4.14 (t, *J* = 5.9 Hz, 1H, Gal-H), 3.46–3.40 (m, 2H, H-3, H-31-1), 3.12–3.06 (m, 2H, H-18, H-31-2), 1.34 (s, 3H, CH_3_), 1.31 (s, 3H, CH_3_), 1.01 (s, 3H, CH_3_), 0.97 (s, 6H, 2 × CH_3_), 0.95 (s, 3H, CH_3_), 0.91 (s, 3H, CH_3_), 0.91 (d, *J* = 6.7 Hz, 6H, H-33, H-34); ^13^C-NMR (150 MHz, pyridine-*d*_5_) *δ*: 177.4, 145.1, 122.7, 107.4, 88.7, 76.8, 75.4, 73.2, 70.3, 62.4, 55.8, 47.9, 47.3, 46.8, 46.5, 42.2, 42.1, 39.8, 39.5, 38.8, 37.0, 34.4, 33.8, 33.1 (C × 2), 30.8, 28.7, 28.2, 27.9, 26.6, 26.1, 23.8 (C × 2), 23.7, 20.4 (C × 2), 18.5, 17.5, 16.9, 15.5; HRMS (ESI): Calcd for [M + Na]^+ ^C_40_H_67_NNaO_7_: 696.4815, found 696.4811.

*28-N-Propargyl oleanolic amide 3-O-β-d-galactopyranoside *(**6b**): White solid, 75% yield; [α]${}_{D}^{25}$: +57.00 (c 0.13, MeOH); ^1^H-NMR (600 MHz, pyridine-*d*_5_) *δ*: 8.08–8.05 (m, 1H, N-H), 5.44 (t, *J* = 3.5 Hz, 1H, H-12), 4.88 (d, *J* = 7.7 Hz, 1H, H-1′), 4.61–4.60 (m, 1H, Gal-H), 4.52–4.46 (m, 3H, Gal-H), 4.43–4.39 (m, 1H, H-31-1), 4.33–4.29 (m, 1H, H-31-2), 4.19 (dd, *J* = 3.2 Hz, 9.3 Hz, 1H, Gal-H), 4.14 (t, *J* = 5.9 Hz, 1H, Gal-H), 3.41 (dd, *J* = 11.8 Hz, 4.4 Hz, 1H, H-3), 3.15 (dd, *J* = 13.6 Hz, 4.4 Hz, 1H, H-18), 3.10 (t, *J* = 2.5 Hz, 1H, H-33), 1.33 (s, 3H, CH_3_), 1.30 (s, 3H, CH_3_), 1.01 (s, 3H, CH_3_), 1.00 (s, 3H, CH_3_), 0.93 (s, 6H, 2 × CH_3_), 0.89 (s, 3H, CH_3_); ^13^C-NMR (150 MHz, pyridine-*d*_5_) *δ*: 177.3, 144.7, 122.9, 107.4, 88.7, 82.1, 76.7, 75.4, 73.2, 71.7, 70.3, 62.4, 55.8, 47.9, 46.7, 46.4, 42.1, 41.7, 39.8, 39.5, 38.7, 37.0, 34.3, 33.5, 33.1, 33.0, 30.8, 29.2, 28.2, 27.8, 26.6, 26.1, 23.8, 23.7, 23.6, 18.4, 17.6, 16.9, 15.5; HRMS (ESI): Calcd for [M + H]^+ ^C_39_H_62_NO_7_: 656.4526, found 656.4516.

*28-N-(3*′*,4*′*-Dimethoxybenzyl) oleanolic amide 3-O-β-d-galactopyranoside *(**6c**): White solid, 73% yield; [α]${}_{D}^{25}$: +20.25 (c 0.13, MeOH); ^1^H-NMR (600 MHz, pyridine-*d*_5_) *δ*: 7.88 (t, *J* = 5.7 Hz, 1H, N-H), 7.12 (d, *J* = 1.8 Hz, 1H, H-2′′), 7.06 (dd, *J* = 8.1 Hz, 1.8 Hz, 1H, H-6′′), 6.92 (d, *J* = 8.2 Hz, 1H, H-5′′), 5.46 (t, *J* = 3.3 Hz, 1H, H-12), 4.88 (d, *J* = 7.8 Hz, 1H, H-1′), 4.79 (dd, *J* = 14.5 Hz, 5.9 Hz, 1H, H-31-1), 4.61–4.56 (m, 2H, H-31-2, Gal-H), 4.52–4.46 (3H, m, Gal-H), 4.19 (dd, *J* = 3.5 Hz, 9.4 Hz, 1H, Gal-H), 4.14 (t, *J* = 6.1 Hz, 1H, Gal-H), 3.77 (s, 3H, Ph-OMe), 3.74 (s, 3H, Ph-OMe), 3.41 (dd, *J* = 11.7 Hz, 4.3 Hz, 1H, H-3), 3.18 (dd, *J* = 13.4 Hz, 4.0 Hz, 1H, H-18), 1.33 (s, 3H, CH_3_), 1.31 (s, 3H, CH_3_), 0.99 (s, 3H, CH_3_), 0.95 (s, 6H, 2 × CH_3_), 0.91 (s, 3H, CH_3_), 0.90 (s, 3H, CH_3_); ^13^C-NMR (150 MHz, pyridine-*d*_5_) *δ*: 177.4, 149.1, 144.9, 133.3, 122.8, 120.5, 112.7, 107.5, 88.7, 76.8, 75.4, 73.2, 70.3, 62.5, 56.0, 55.8 (C × 2), 48.0, 46.8, 46.5, 43.4, 42.2, 42.0, 39.8, 39.5, 38.7, 37.0, 34.4, 33.8, 33.1, 33.0, 30.8, 28.2, 27.9, 26.6, 26.1, 23.8 (C × 2), 23.7, 18.4, 17.4, 17.0, 15.5; HRMS (ESI): Calcd for [M + Na]^+ ^C_45_H_69_NNaO_9_: 790.4870, found 790.4860.

*28-N-Isobutyl ursolic amide 3-O-β-d-glucopyranoside *(**7a**): White solid, 85% yield; [α]${}_{D}^{25}$: +27.00 (c 0.13, MeOH); ^1^H-NMR (600 MHz, pyridine-*d*_5_) *δ*: 7.18 (t, *J* = 5.8 Hz, 1H, N-H), 5.47 (t, *J* = 3.4 Hz, 1H, H-12), 4.97 (d, *J* = 7.7 Hz, 1H, H-1′), 4.62 (dd, *J* = 11.7 Hz, 2.4 Hz, 1H, Glu-H), 4.43 (dd, *J* = 11.7 Hz, 5.5 Hz, 1H, Glu-H), 4.29–4.22 (m, 2H, Glu-H), 4.08–4.02 (m, 2H, Glu-H), 3.44 (dd, *J* = 11.9 Hz, 4.5 Hz, 1H, H-3), 3.35–3.31 (m, 1H, H-31-1), 3.18–3.14 (m, 1H, H-31-2), 2.40 (d, *J* = 10.6 Hz, 1H, H-18), 1.35 (s, 3H, CH_3_), 1.24 (s, 3H, CH_3_), 1.04 (s, 3H, CH_3_), 0.99 (s, 3H, CH_3_), 0.97 (d, J = 6.5 Hz, 3H, CH_3_), 0.95 (s, 3H, CH_3_), 0.92–0.89 (m, 9H, 3 × CH_3_); ^13^C-NMR (150 MHz, pyridine-*d*_5_) *δ*: 177.3, 139.8, 125.7, 106.9, 88.9, 78.7, 78.2, 75.8, 71.9, 63.1, 55.8, 53.7, 47.9 (C × 2), 47.4, 42.6, 40.0, 39.9, 39.5, 39.3, 38.9, 38.2, 36.9, 33.4, 31.2, 28.6, 28.3 (C × 2), 26.6, 24.9, 23.7, 23.6, 21.3, 20.5 (C × 2), 18.4, 17.4 (C × 2), 17.0, 15.6; HRMS (ESI): Calcd for [M + Na]^+^ C_40_H_67_NNaO_7_: 696.4815, found 696.4807.

*28-N-Propargyl ursolic amide 3-O-β-d-glucopyranoside *(**7b**): White solid, 72% yield; [α]${}_{D}^{25}$: +24.00 (c 0.13, MeOH); ^1^H-NMR (600 MHz, pyridine-*d*_5_) *δ*: 7.85 (t, *J* = 5.3 Hz, 1H, N-H), 5.45 (t, *J* = 3.3 Hz, 1H, H-12), 4.97 (d, *J* = 7.8 Hz, 1H, H-1′), 4.62 (dd, *J* = 11.5 Hz, 1.9 Hz, 1H, Glu-H), 4.44–4.35 (m, 2H, H-31-1, Glu-H), 4.33–4.22 (m, 3H, H-31-2, Glu-H), 4.08–4.02 (m, 2H, Glu-H), 3.43 (dd, *J* = 11.9 Hz, 4.4 Hz, 1H, H-3), 3.10 (t, *J* = 2.3 Hz, 1H, H-33), 2.44 (d, *J* = 10.8 Hz, 1H, H-18), 1.35 (s, 3H, CH_3_), 1.23 (s, 3H, CH_3_), 1.03 (s, 3H, CH_3_), 1.02 (s, 3H, CH_3_), 0.97 (d, *J* = 6.5 Hz, 3H, CH_3_), 0.94 (s, 3H, CH_3_), 0.89 (s, 3H, CH_3_); ^13^C-NMR (150 MHz, pyridine-*d*_5_) *δ*: 177.2, 139.4, 126.0, 106.9, 88.9, 81.9, 78.7, 78.3, 75.8, 71.9 (C × 2), 63.1, 55.8, 53.3, 47.9, 47.8, 42.5, 40.0, 39.7, 39.5, 39.3, 38.9, 37.8, 36.8, 33.3, 31.1, 29.2, 28.3, 26.6, 24.8, 23.8, 23.6, 21.3, 18.4, 17.6, 17.4, 17.0, 15.6; HRMS (ESI): Calcd for [M + H]^+^ C_39_H_62_NO_7_: 656.4526, found 656.4520.

*28-N-(3*′*,4*′*-Dimethoxybenzyl) ursolic amide 3-O-β-d-glucopyranoside* (**7c**): White solid, 74% yield; [α]${}_{D}^{25}$: +7.5 (c 0.13, MeOH); ^1^H-NMR (600 MHz, pyridine-*d*_5_) *δ*: 7.71 (t, *J* = 5.6 Hz, 1H, N-H), 7.11 (d, *J* = 1.8 Hz, 1H, H-2′′), 7.05 (dd, *J* = 8.1 Hz, 1.9 Hz, 1H, H-6′′), 6.94 (d, *J* = 8.2 Hz, 1H, H-5′′), 5.45 (t, *J* = 3.3 Hz, 1H, H-12), 4.98 (d, *J* = 7.7 Hz, 1H, H-1′), 4.71 (dd, *J* = 14.4 Hz, 5.6 Hz, 1H, H-31-1), 4.64–4.60 (m, 2H, H-31-2, Glu-H), 4.44 (dd, *J* = 11.8 Hz, 5.6 Hz, 1H, Glu-H), 4.29–4.23 (m, 2H, Glu-H), 4.09–4.03 (m, 2H, Glu-H), 3.78 (s, 3H, Ph-OMe), 3.75 (s, 3H, Ph-OMe), 3.44 (dd, *J* = 11.8 Hz, 4.4 Hz, 1H, H-3), 2.45 (d, *J* = 10.7 Hz, 1H, H-18), 1.35 (s, 3H, CH_3_), 1.25 (s, 3H, CH_3_), 1.03 (s, 3H, CH_3_), 0.97 (d, *J* = 6.4 Hz, 3H, CH_3_), 0.95 (s, 3H, CH_3_), 0.91 (s, 6H, 2 × CH_3_); ^13^C-NMR (150 MHz, pyridine-*d*_5_) *δ*: 177.2, 149.1, 139.7, 133.1, 125.8, 120.6, 112.8, 112.6, 106.9, 88.9, 78.7, 78.3, 75.8, 71.9, 63.1, 56.0, 55.9, 55.8, 53.6, 47.9, 47.8, 43.5, 42.5, 40.0, 39.8, 39.5, 39.3, 38.9, 38.1, 36.9, 33.4, 31.2, 28.4, 28.3, 26.6, 24.9, 23.8, 23.6, 21.3, 21.2, 18.4, 17.4, 17.0, 15.6; HRMS (ESI): Calcd for [M + Na]^+ ^C_45_H_69_NNaO_9_: 790.4870, found 790.4863.

*28-N-Isobutyl ursolic amide 3-O-β-d-galactopyranoside *(**8a**): White solid, 89% yield; [α]${}_{D}^{25}$: +41.25 (c 0.13, MeOH); ^1^H-NMR (600 MHz, pyridine-*d*_5_) *δ*: 7.19 (t, *J* = 5.9 Hz, 1H, N-H), 5.47 (t, *J* = 3.4 Hz, 1H, H-12), 4.89 (d, *J* = 7.7 Hz, 1H, H-1′), 4.60–4.59 (m, 1H, Gal-H), 4.52–4.46 (3H, m, Gal-H), 4.19 (dd, *J* = 3.4 Hz, 9.5 Hz, 1H, Gal-H), 4.14 (t, *J* = 6.0 Hz, 1H, Gal-H), 3.44 (dd, *J* = 11.8 Hz, 4.2 Hz, 1H, H-3), 3.35–3.31 (m, 1H, H-31-1), 3.17–3.13 (m, 1H, H-31-2), 2.41 (d, *J* = 10.8 Hz, 1H, H-18), 1.34 (s, 3H, CH_3_), 1.25 (s, 3H, CH_3_), 1.01 (s, 3H, CH_3_), 0.99 (s, 3H, CH_3_), 0.97 (d, *J* = 6.6 Hz, 3H, CH_3_), 0.95 (s, 3H, CH_3_), 0.92–0.89 (m, 9H, 3 × CH_3_); ^13^C-NMR (150 MHz, pyridine-*d*_5_) *δ*: 177.3, 139.8, 125.7, 107.5, 88.9, 76.8, 75.4, 73.1, 70.2, 62.4, 55.8, 53.6, 47.9 (C × 2), 47.4, 42.6, 40.0, 39.9, 39.5, 39.3, 38.9, 38.2, 36.9, 33.4, 31.2, 28.6, 28.3, 28.2, 26.6, 24.9, 23.7, 23.6, 21.3, 20.5, 20.4, 18.4, 17.4 (C × 2), 16.9, 15.6; HRMS (ESI): Calcd for [M + Na]^+ ^C_40_H_67_NNaO_7_: 696.4815, found 696.4809.

*28-N-Propargyl ursolic amide 3-O-β-d-galactopyranoside *(**8b**): White solid, 74% yield; [α]${}_{D}^{25}$: +36.00 (c 0.13, MeOH); ^1^H-NMR (600 MHz, pyridine-*d*_5_) *δ*: 7.84 (t, *J* = 5.4 Hz, 1H, N-H), 5.45 (t, *J* = 3.3 Hz, 1H, H-12), 4.89 (d, *J* = 7.7 Hz, 1H, H-1′), 4.60–4.59 (m, 1H, Gal-H), 4.52–4.46 (m, 3H, Gal-H), 4.40–4.28 (m, 2H, H-31), 4.19 (dd, *J* = 3.4 Hz, 9.4 Hz, 1H, Gal-H), 4.13 (t, *J* = 6.2 Hz, 1H, Gal-H), 3.43 (dd, *J* = 11.8 Hz, 4.5 Hz, 1H, H-3), 3.10 (t, *J* = 2.4 Hz, 1H, H-33), 2.44 (d, J = 10.8 Hz, 1H, H-18), 1.34 (s, 3H, CH_3_), 1.24 (s, 3H, CH_3_), 1.02 (s, 3H, CH_3_), 1.00 (s, 3H, CH_3_), 0.97 (d, *J* = 6.5 Hz, 3H, CH_3_), 0.94 (s, 3H, CH_3_), 0.90 (s, 3H, CH_3_); ^13^C-NMR (150 MHz, pyridine-*d*_5_) *δ*: 177.2, 139.4, 126.0, 107.5, 88.8, 81.9, 76.8, 75.4, 73.1, 71.9, 70.3, 62.5, 55.9, 53.3, 47.9, 47.8, 42.5, 40.0, 39.8, 39.5, 39.3, 38.9, 37.8, 36.8, 33.3, 31.1, 29.2, 28.3, 26.7, 24.8, 23.8, 23.6, 21.3, 18.4, 17.6, 17.4, 17.0, 15.6; HRMS (ESI): Calcd for [M + H]^+ ^C_39_H_62_NO_7_: 656.4526, found 656.4516.

*28-N-(3*′*,4*′*-Dimethoxybenzyl) ursolic amide 3-O-β-d-galactopyranoside *(**8c**): White solid, 69% yield; [α]${}_{D}^{25}$: +8.25 (c 0.13, MeOH); ^1^H-NMR (600 MHz, pyridine-*d*_5_) *δ*: 7.70 (t, *J* = 5.8 Hz, 1H, N-H), 7.11 (d, *J* = 1.8 Hz, 1H, H-2′′), 7.05 (dd, *J* = 8.2 Hz, 2.0 Hz, 1H, H-6′′), 6.94 (d, *J* = 8.2 Hz, 1H, H-5′′), 5.45 (t, *J* = 3.3 Hz, 1H, H-12), 4.89 (d, *J* = 7.7 Hz, 1H, H-1′), 4.71 (dd, *J* = 14.5 Hz, 5.9 Hz, 1H, H-31-1), 4.62–4.59 (m, 2H, H-31-2, Gal-H), 4.53–4.46 (3H, m, Gal-H), 4.19 (dd, *J* = 3.3 Hz, 9.4 Hz, 1H, Gal-H), 4.14 (t, *J* = 5.9 Hz, 1H, Gal-H), 3.78 (s, 3H, Ph-OMe), 3.75 (s, 3H, Ph-OMe), 3.44 (dd, *J* = 11.9 Hz, 4.4 Hz, 1H, H-3), 2.45 (d, *J* = 10.7 Hz, 1H, H-18), 1.34 (s, 3H, CH_3_), 1.25 (s, 3H, CH_3_), 1.00 (s, 3H, CH_3_), 0.97 (d, *J* = 6.5 Hz, 3H, CH_3_), 0.95 (s, 3H, CH_3_), 0.93 (s, 3H, CH_3_), 0.91 (s, 3H, CH_3_); ^13^C-NMR (150 MHz, pyridine-*d*_5_) *δ*: 177.2, 149.1, 139.7, 133.1, 125.8, 120.6, 112.8, 112.6, 107.5, 88.8, 76.8, 75.4, 73.2, 70.3, 62.5, 56.0, 55.9, 53.6, 47.9, 47.8, 43.5, 42.5, 40.0, 39.8, 39.5, 39.3, 38.9, 38.1, 36.9, 33.4, 31.2, 28.4, 28.3, 26.7, 24.9, 23.8, 23.6, 21.3, 18.4, 17.5, 17.4, 17.0, 15.6; HRMS (ESI): Calcd for [M + Na]^+ ^C_45_H_69_NNaO_9_: 790.4870, found 790.4860.

The spectrograms of the compounds **5a–c**, **6a–c**, **7a–c** and **8a–c** were shown in Electronic Supplementary Material (ESM).

### 3.3. Evaluation of the Biological Activity

#### 3.3.1. Cell Culture and Treatment

The H9c2 rat myocardial cell line was obtained from the Chinese Academy of Sciences Cell Bank (Shanghai, China) and cultured as previously described [20]. Briefly, H9c2 cells were cultured in high glucose DMEM supplemented with 10% (*v*/*v*) fetal bovine serum, 1% penicillin/streptomycin (*v*/*v*), and 2 mM l-glutamine. The cells were maintained in a humidified incubator with 95% air and 5% CO_2_ at 37 °C.

The cells were subcultured after reaching 70~80% confluence. Three sets of experiments were performed: (1) control cells; (2) cells treated with H_2_O_2_ (450 μM) for 1 h; (3) cells pretreated with oleanolic acid 3-*O*-*β*-d-glucuronopyranoside derivatives (0.1 μg/mL) for 12 h, then the medium was removed and the cells were treated with H_2_O_2_ (450 μM) for 1 h. For all of the experiments, the cells were plated at an appropriate density according to the experimental design and were grown for 36 h before experimentation.

#### 3.3.2. Determination of Cell Viability

Cell viability was determined by the 3-(4,5-dimethylthiazol-2-yl)-2,5-diphenyl tetrazolium (MTT) assay as previously described [20]. Briefly, H9c2 cells were plated in 96-well plates at a density of 1 × 10^4^ cells/well and incubated overnight. After the designated treatment, 20 μL of MTT (5 mg/mL) was added to each well and cells were incubated at 37 °C for 4 h. Then, the culture medium with MTT was abandoned and the colored formazan crystals were dissolved in 100 μL dimethyl sulfoxide (DMSO). The absorption values were measured at 570 nm using a microplate reader (TECAN Infinite M1000, Grödig, Austria). The viability of H9c2 cells in each well is presented as a percentage of control cells.

#### 3.3.3. Statistic Analysis

The data are expressed as mean ± SD. Comparisons were performed by Student’s *t*-test or one-way ANOVA followed by Tukey’s multiple comparison test with Prism 5.00 software. Statistical significance was set at *p* < 0.05. All data are the results of at least three independent experiments.

## 4. Conclusions

In summary, a new series of oleanolic acid 3-*O*-*β*-d-glucuronopyranoside derivatives were designed, synthesized, and evaluated for their cardiovascular protective effect. Some of the synthesized compounds showed potent protective activity against H_2_O_2_-induced injury in H9c2 cells. Particularly, compound **8a** exhibited a greater potential protective effect than the positive control oleanolic acid 3-*O*-*β*-d-glucuronopyranoside. Preliminary SAR analysis has shown that the isobutyl group of the amide derivatives had a good impact on the protective effect.

## Supplementary Materials

Supplementary materials are available online, Figures S1–S24: ^1^H-NMR and ^13^C-NMR of compound **5a**–**c**, **6a**–**c**, **7a**–**c** and **8a**–**c**.

## References

[1] Chaudhari K., Hamad B., Syed B.A. (2014). Antithrombotic drugs market. Nat. Rev. Drug Discov..

[2] Li C.L., Dong M.H., Ren Y.J., Li L.H. (2015). Design, synthesis, biological evaluation and molecular docking of novel dabigatran derivatives as potential thrombin inhibitors. RSC Adv..

[3] Megson L.L., Whitfield P.D., Zabetakis L. (2016). Lipids and cardiovascular disease: Where does dietary intervention sit alongside statin therapy?. Food Funct..

[4] Brown A., Reynolds L.R., Bruemmer D. (2010). Intensive glycemic control and cardiovascular disease: An update. Nat. Rev. Cardiol..

[5] Morrish N.J., Wang S.L., Stevens L.K., Fuller J.H., Keen H. (2001). Mortality and causes of death in the WHO multinational study of vascular disease in diabetes. Diabetologia.

[6] Zhang J.F., Fan S.R., Mao Y.G., Ji Y., Jin L.Q., Lu J.X., Chen X.M. (2016). Cardiovascular protective effect of polysaccharide from Ophiopogon japonicus in diabetic rats. Int. J. Biol. Macromol..

[7] Baranov A.I. (1982). Medicinal uses of ginseng and related plants in the Soviet Union: Recent trends in the Soviet literature. J. Ethnopharmacol..

[8] Jia Y.L., Dong X.Y., Zhou P.F., Liu X.H., Pan L.L., Xin H., Zhu Y.Z., Wang Y. (2012). The synthesis and biological evaluation of novel Danshensu-cysteine analog conjugates as cardiovascular-protective agents. Eur. J. Med. Chem..

[9] Nhiem N.X., Lim H.Y., Kiem P.V., Minh C.V., Thu V.K., Tai B.H., Quang T.H., Song S.B., Kim Y.H. (2011). Oleanane-type triterpene saponins from the bark of Aralia elata and their NF-kappa B inhibition and PPAR activation signal pathway. Bioorg. Med. Chem. Lett..

[10] Sun G.B., Xu H.B., Wen F.C., Zhang W., Ding T., Sun X.B. (2006). Protective effects of aralosides on cultured myocardial cells subjected to anoxia/reoxygenation injury. Chin. Pharmacol. Bull..

[11] Wen F.C., Xu H.B., Zhang W., Ding T., Sun X.B. (2005). Studies on antimyocardiac ischemia of Aralia elata saponin. World Sci. Technol. Mod. Tradit. Chin. Med. Mater. Med..

[12] Deng H.W., Li Y.J., Shen N., Chen X., Zhou Z.C. (1988). Protective effect of aralosides of Aralia elataon experimental myocardial ischemia of rats. Chin. J. Pharmacol..

[13] Wang M., Xu X.D., Xu H.B., Wen F.C., Zhang X.P., Sun H., Yao F., Sun G.B., Sun X.B. (2014). Effect of the total saponins of Aralia elata (Miq) Seem on cardiac contractile function and intracellular calcium cycling regulation. J. Ethnopharmacol..

[14] Lee J.H., Ha Y.W., Jeong C.S., Kim Y.S., Park Y. (2009). Isolation and tandem mass fragmentations of an anti-inflammatory compound from Aralia elata. Arch. Pharm. Res..

[15] Wang Z., Song S., Lu H., Chen G., Xu S., Sagara Y., Kitaoka N., Manabe M., Kodama H. (2003). Effect of three triterpenoid compounds isolated from root bark of Aralia elataon stimulus-induced superoxide generation and tyrosyl phosphorylation and translocation of p47(phox) and p67(phox) to cell membrane in human neutrophil. Clin. Chim. Acta.

[16] Wang M., Meng X.B., Yu Y.L., Sun G.B., Xu X.D., Zhang X.P., Dong X., Ye J.X., Xu H.B., Sun Y.F. (2014). Elatoside C protects against hypoxia/reoxygenation-induced apoptosis in H9c2 cardiomyocytes through the reduction of endoplasmic reticulum stress partially depending on STAT3 activation. Apoptosis.

[17] Wang M., Sun G.B., Zhang J.Y., Luo Y., Yu Y.L., Xu X.D., Meng X.B., Zhang M.D., Lin W.B., Sun X.B. (2015). Elatoside C protects the heart from ischaemia/reperfusion injury through the modulation of oxidative stress and intracellular Ca^2+^ homeostasis. Int. J. Cardiol..

[18] Wang M., Tian Y., Du Y.Y., Sun G.B., Xu X.D., Jiang H., Xu H.B., Meng X.B., Zhang J.Y., Ding S.L. (2017). Protective effects of Araloside C against myocardial ischaemia/reperfusion injury: Potential involvement of heat shock protein 90. J. Cell. Mol. Med..

[19] Tian Y., Wang S., Shang H., Wang M., Sun G.B., Xu X.D., Sun X. (2017). The proteomic profiling of calenduloside E targets in HUVEC: Design, synthesis and application of biotinylated probe BCEA. RSC Adv..

[20] Sun G.B., Sun X., Wang M., Ye J.X., Si J.Y., Xu H.B., Meng X.B., Qin M., Sun J., Wang H.W. (2012). Oxidative stress suppression by luteolin-induced heme oxygenase-1 expression. Toxicol. Appl. Pharmacol..

