# Supplementary material for: Semisynthesis and Biological Evaluation of Oleanolic Acid 3-O-β-d-Glucuronopyranoside Derivatives for Protecting H9c2 Cardiomyoblasts against H2O2-Induced Injury
Source: Molecules. 2018 Jan 10;23(1):44. doi: 10.3390/molecules23010044 (PMC5943939; doi:10.3390/molecules23010044)
Supplement: Supplementary file 1 [file molecules-23-00044-s001.pdf]

Semisynthesis and Biological Evaluation of Oleanolic Acid 3-*O*-*β*-D-Glucuronopyranoside Derivatives for Protecting H9c2 Cardiomyoblasts against H_2O_2-Induced Injury

Yu Tian [1], Zhonghao Sun [1], Wenqian Wang [2], Hai Shang [1], Baoqi Wang [3], Di Deng [3], Guoxu Ma [1], Haifeng Wu [1], Nailiang Zhu [1], Xudong Xu [1,*], Guibo Sun [1,*] and Xiaobo Sun [1,*]

[1] Beijing Key Laboratory of Innovative Drug Discovery of Traditional Chinese Medicine (Natural Medicine) and Translational Medicine; Key Laboratory of Bioactive Substances and Resources Utilization of Chinese Herbal Medicine, Ministry of Education; Key Laboratory of Efficacy Evaluation of Chinese Medicine against Glycolipid Metabolic Disorders, State Administration of Traditional Chinese Medicine; Zhong guan cun Open Laboratory of the Research and Development of Natural Medicine and Health Products; Institute of Medicinal Plant Development, Chinese Academy of Medical Sciences & Peking Union Medical College, Beijing 100193, China; ytian@implad.ac.cn (Y.T.); sun_zhonghao@126.com (Z.-H.S.); hshang@implad.ac.cn (H.S.); mgxfl8785@163.com (G.-X.M.); hfwu@implad.ac.cn (H.-F.W.); zhu13liang@126.com(N.-L.Z.)

[2] Tianjin Institute of Pharmaceutical Research, Tianjin 300193, China; wangwq@tjipr.com

[3] Center of Research and Development on Life Sciences and Environment Sciences, Harbin University of Commerce, Harbin 150076, Heilongjiang, China; 13115539201@163.com (B.-Q.W); 18745112039@163.com (D.D.)

* Correspondence: xdxu@implad.ac.cn (X.-D.X.), gbsun@implad.ac.cn (G.-B.S.), sunsubmit@163.com (X.-B.S.); Tel./Fax: +86-10-5783-3290 (X.-D.X.)

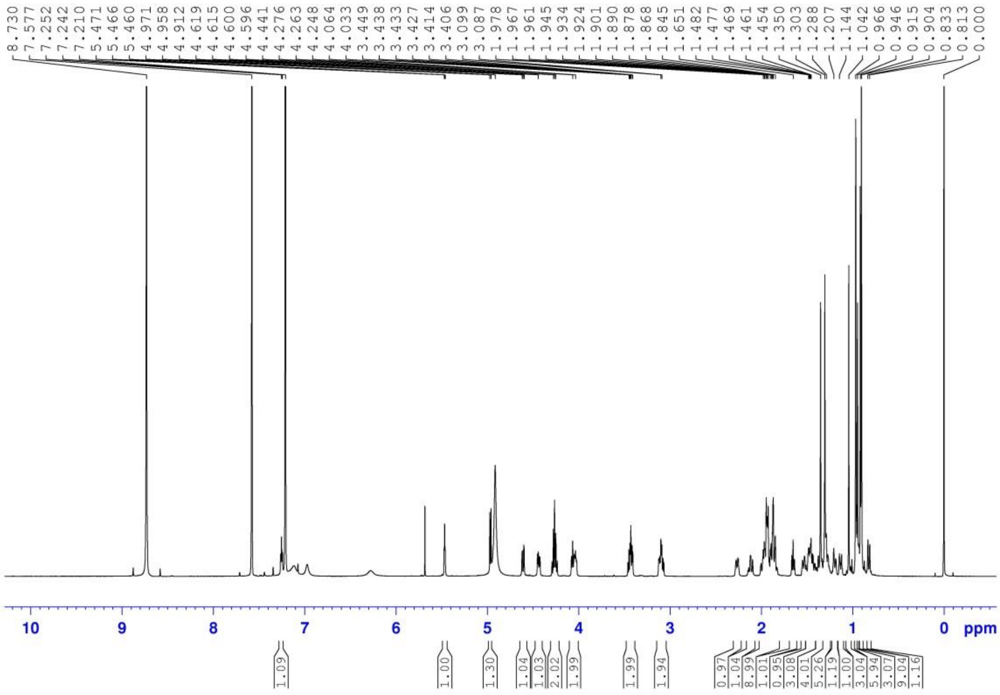

Figure S1 ^{1}H NMR of compound **5a**

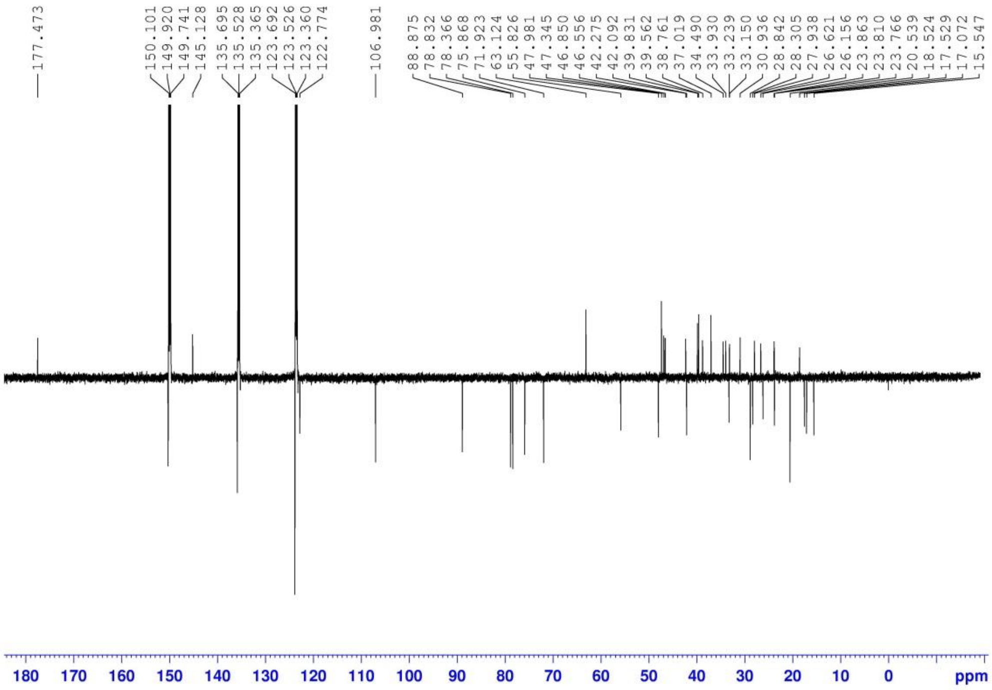

Figure S2 ^{13}C NMR of compound **5a**

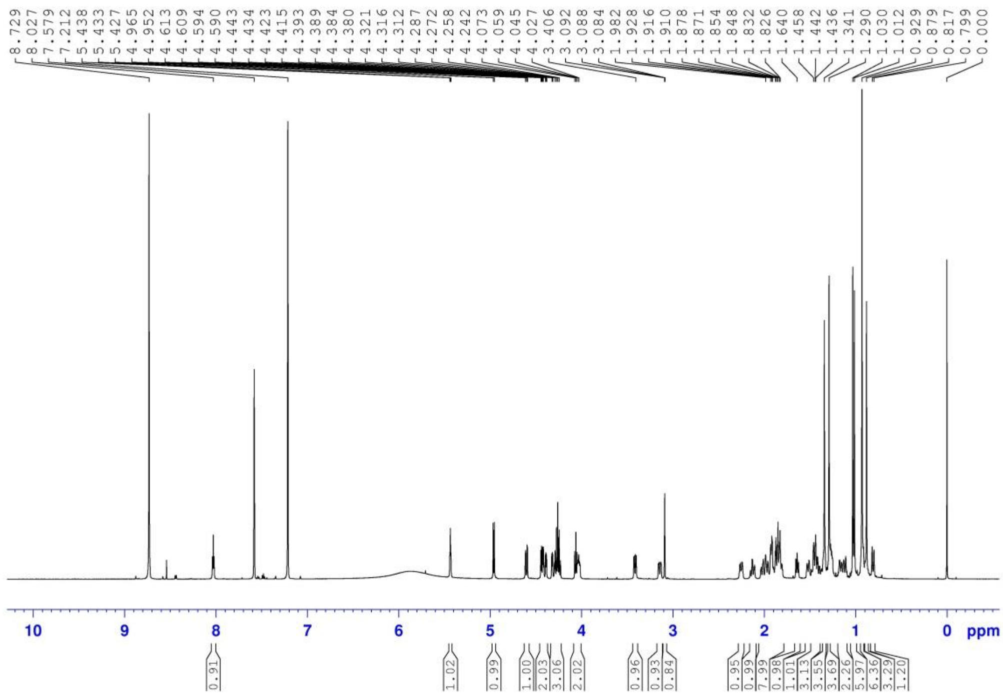

Figure S3 ^{1}H NMR of compound **5b**

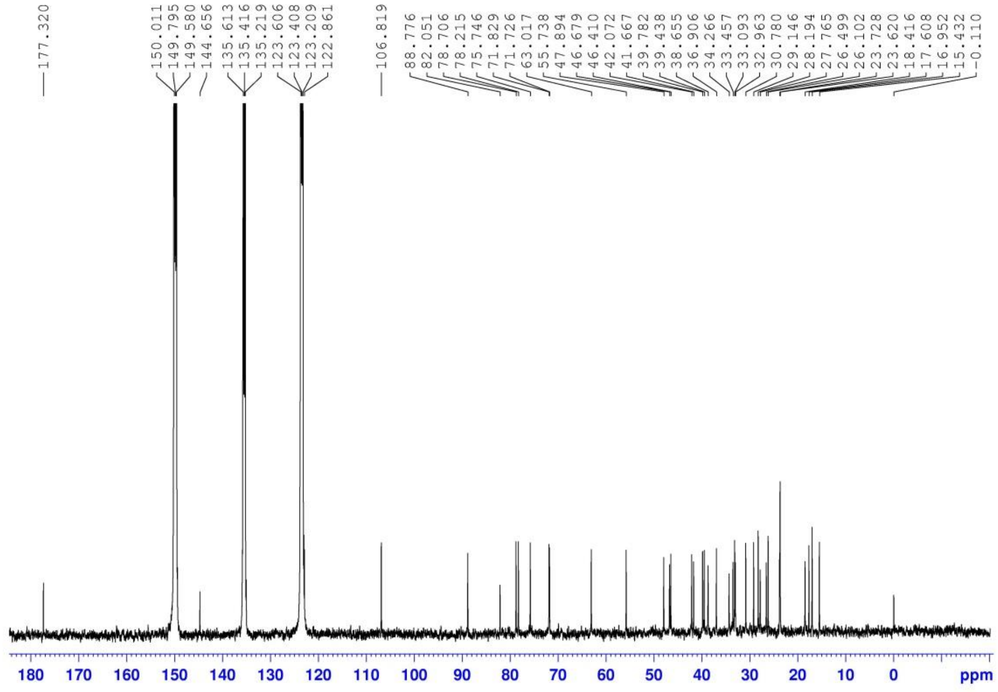

Figure S4 ^{13}C NMR of compound **5b**

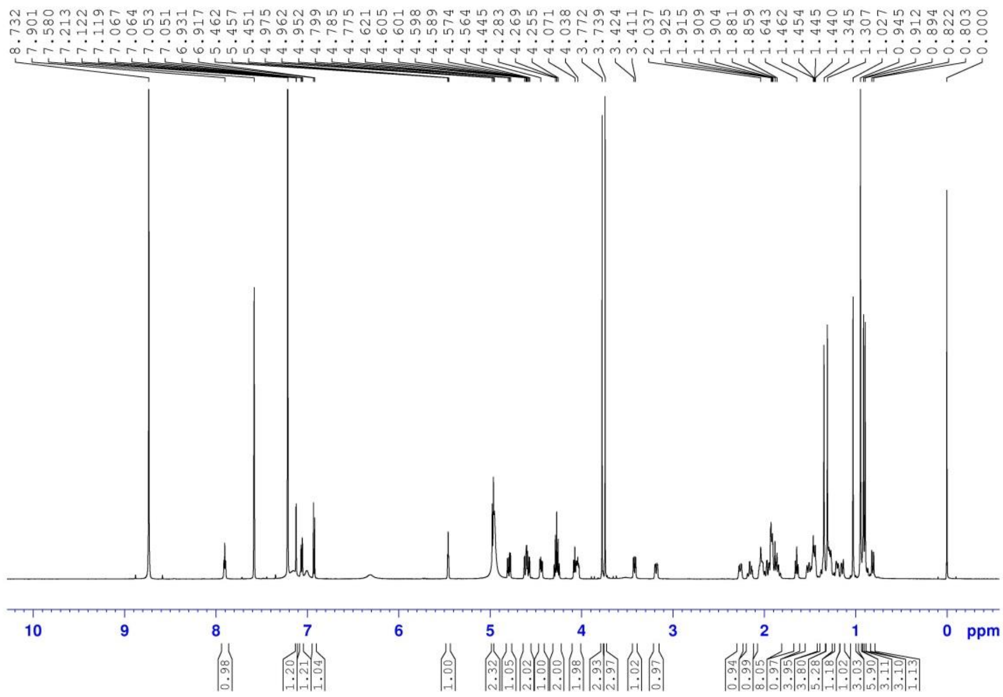

Figure S5 ^{1}H NMR of compound **5c**

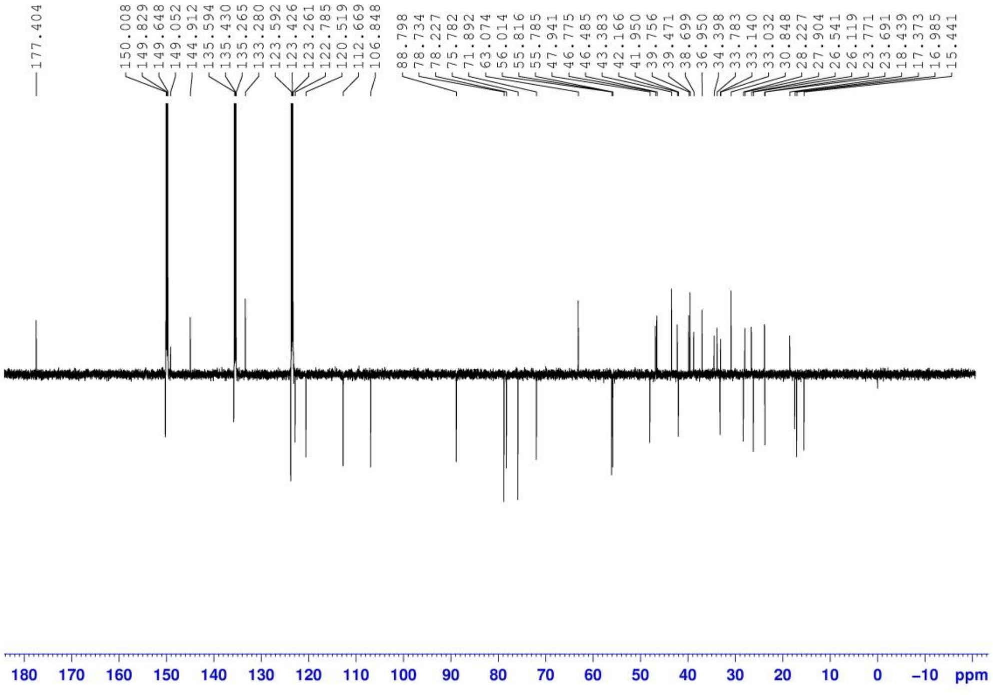

Figure S6 ^{13}C NMR of compound **5c**

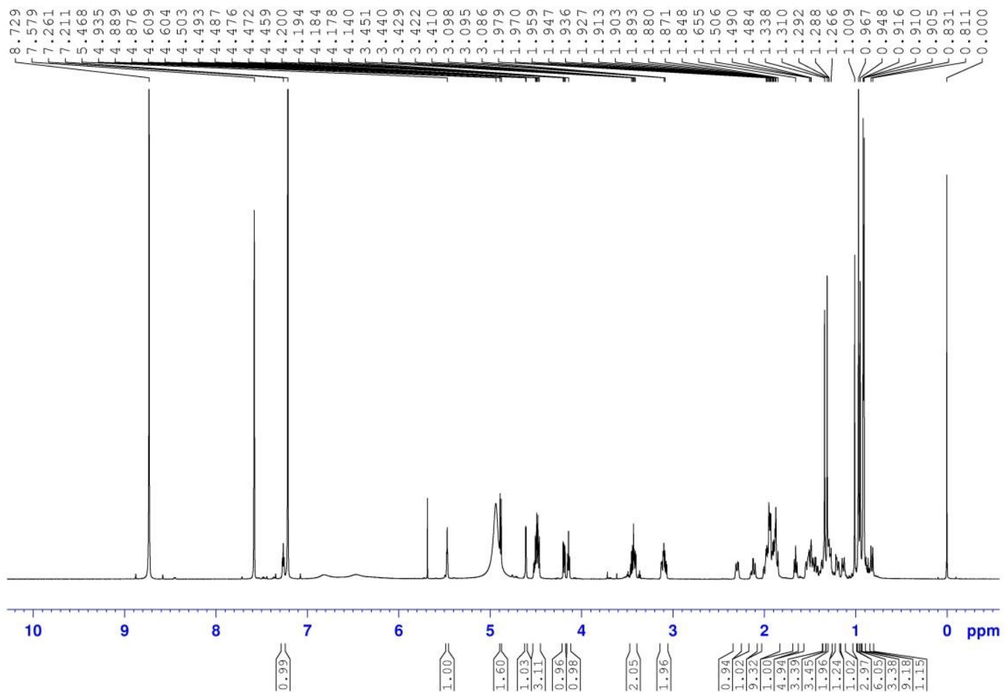

Figure S7 ^{1}H NMR of compound **6a**

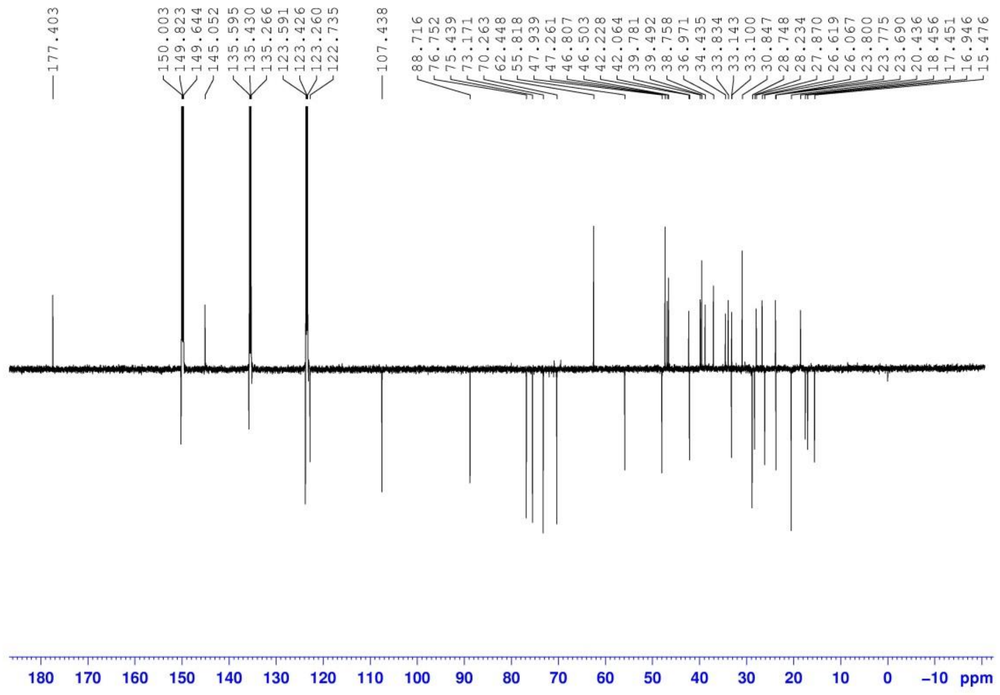

Figure S8 ^{13}C NMR of compound **6a**

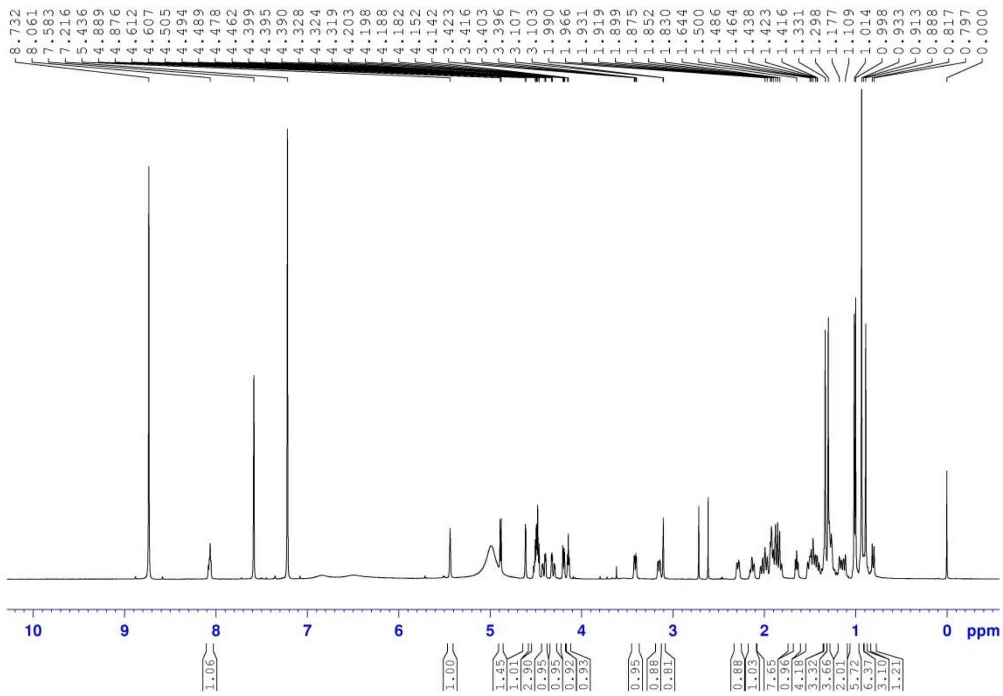

Figure S9 ^{1}H NMR of compound **6b**

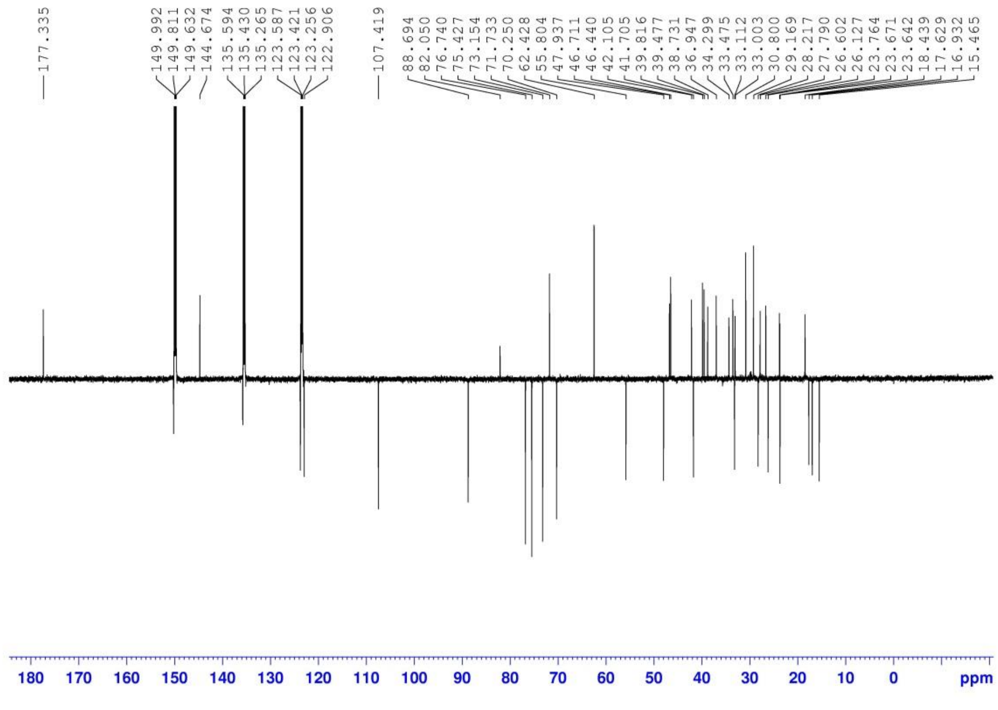

Figure S10 ^{13}C NMR of compound **6b**

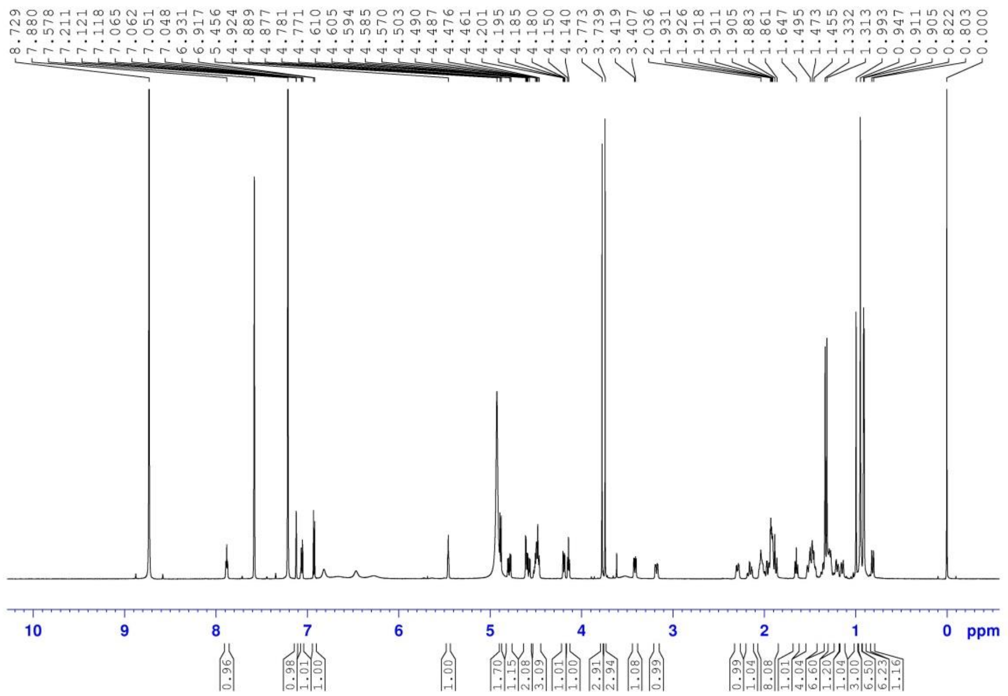

Figure S11 ^{1}H NMR of compound **6c**

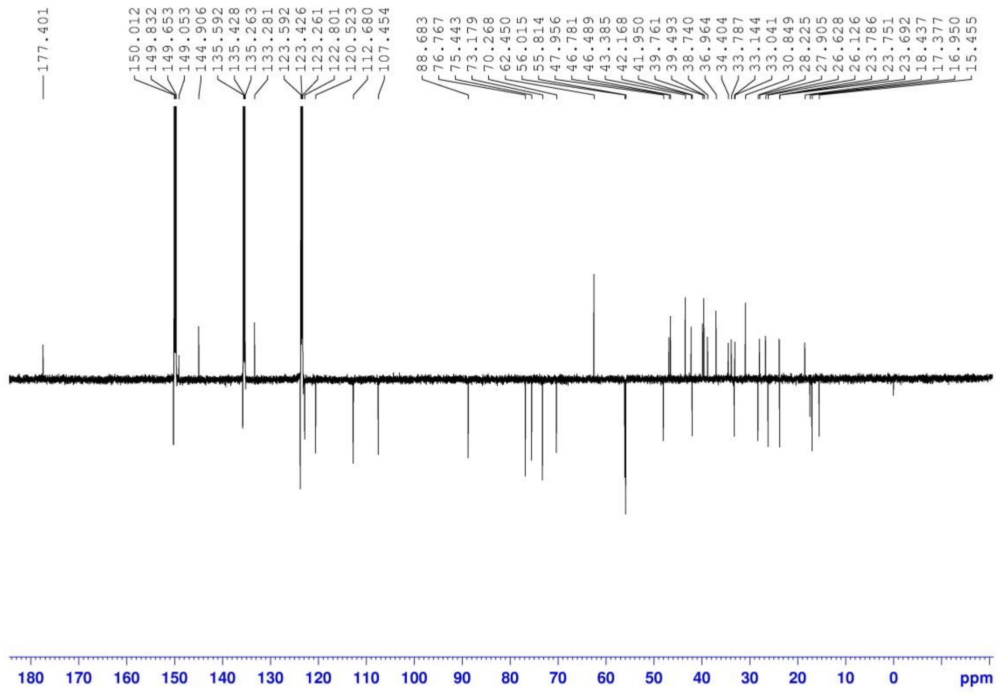

Figure S12 ^{13}C NMR of compound **6c**

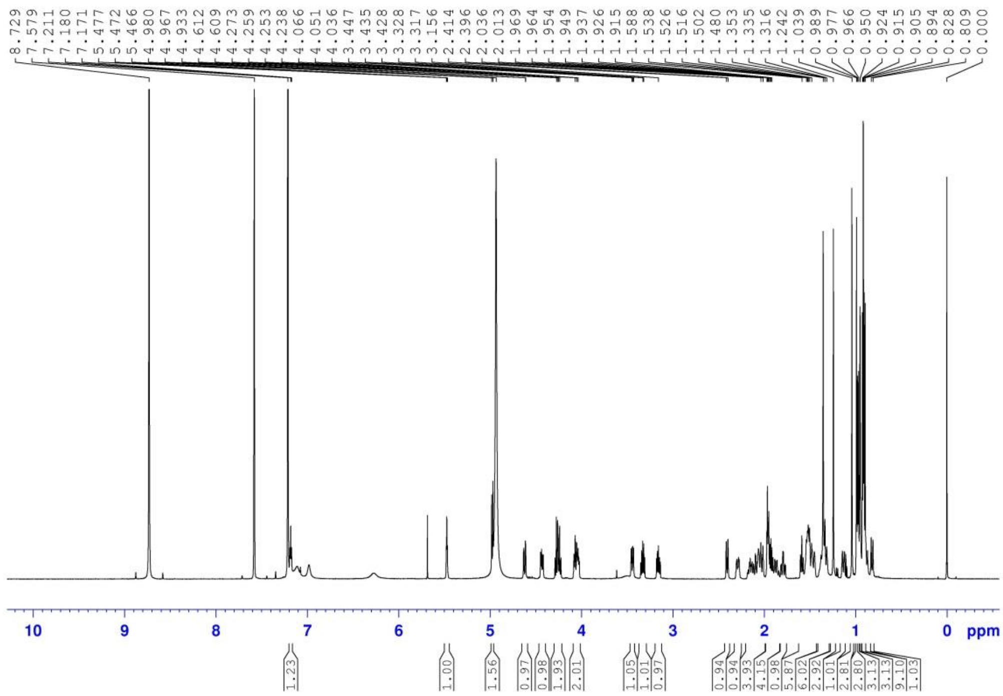

Figure S13 ^{1}H NMR of compound **7a**

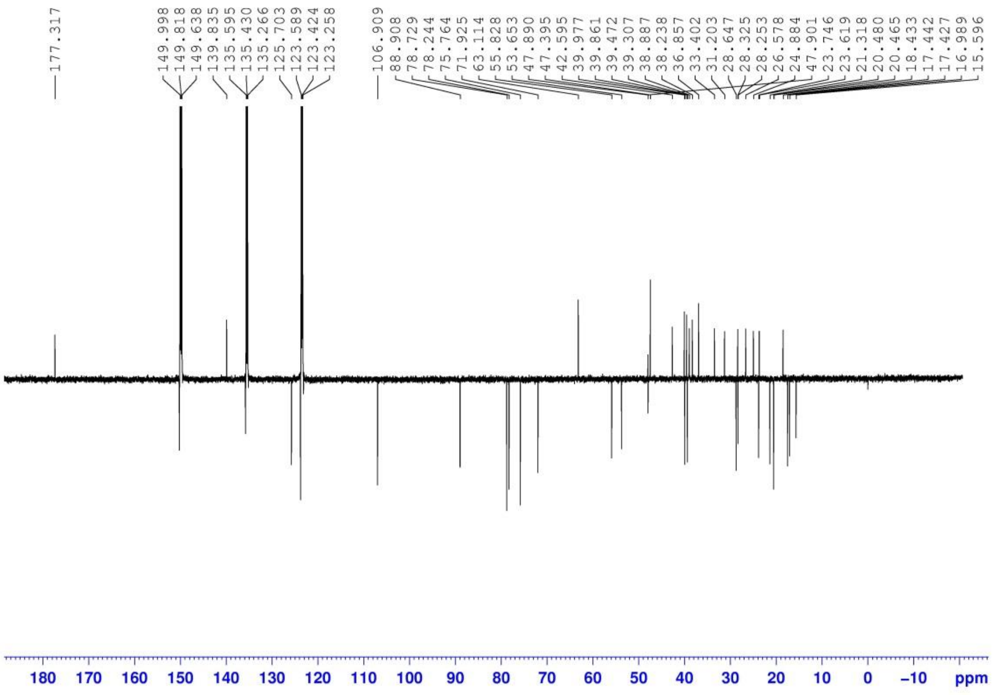

Figure S14 ^{13}C NMR of compound **7a**

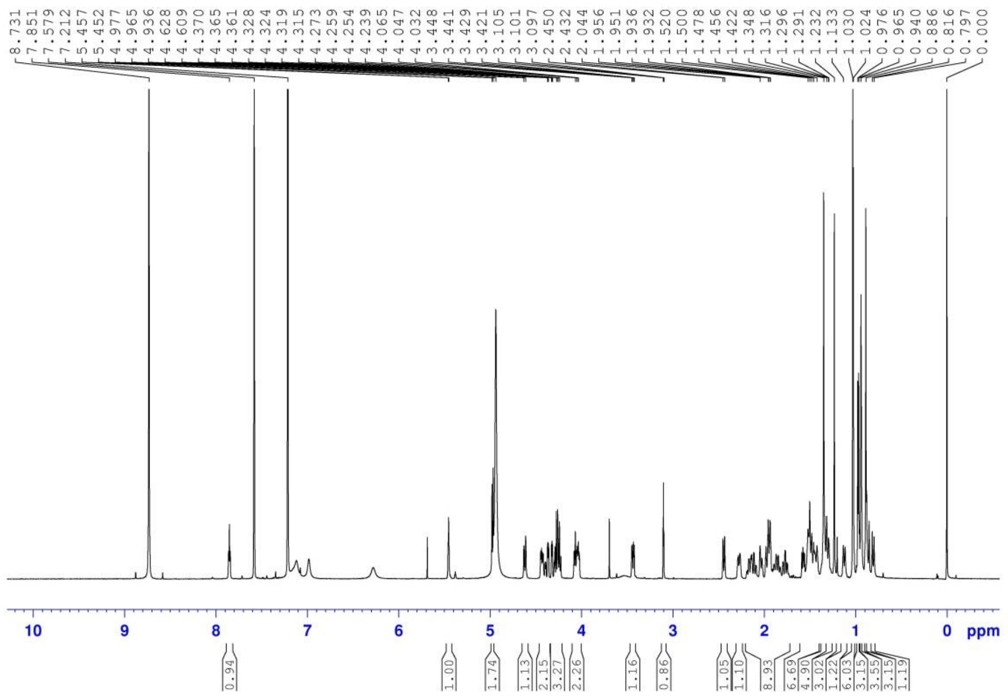

Figure S15 ^{1}H NMR of compound **7b**

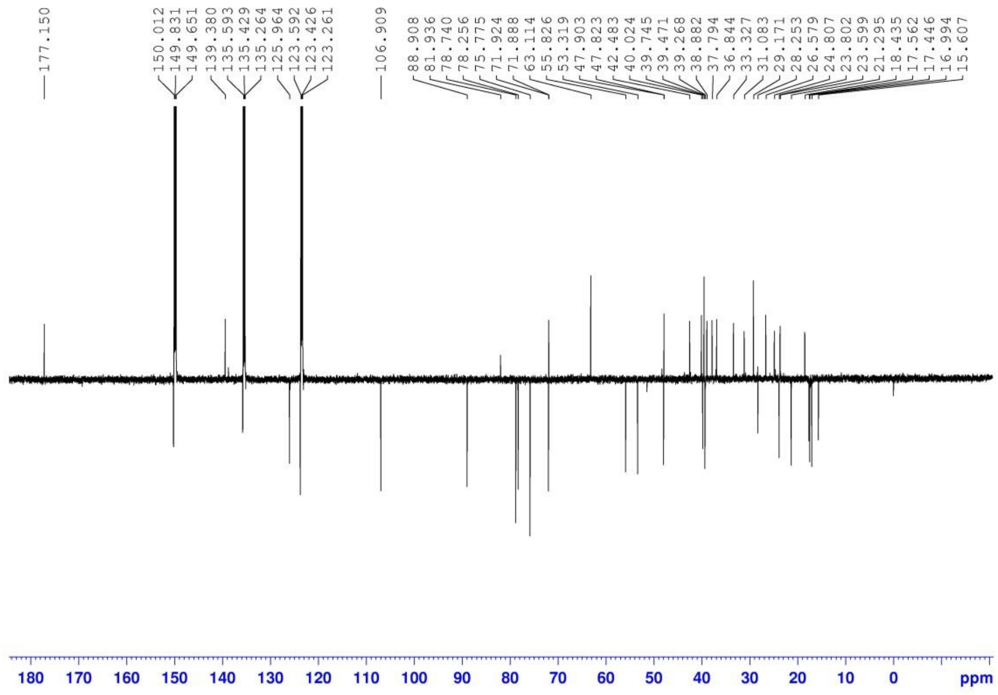

Figure S16 ^{13}C NMR of compound **7b**

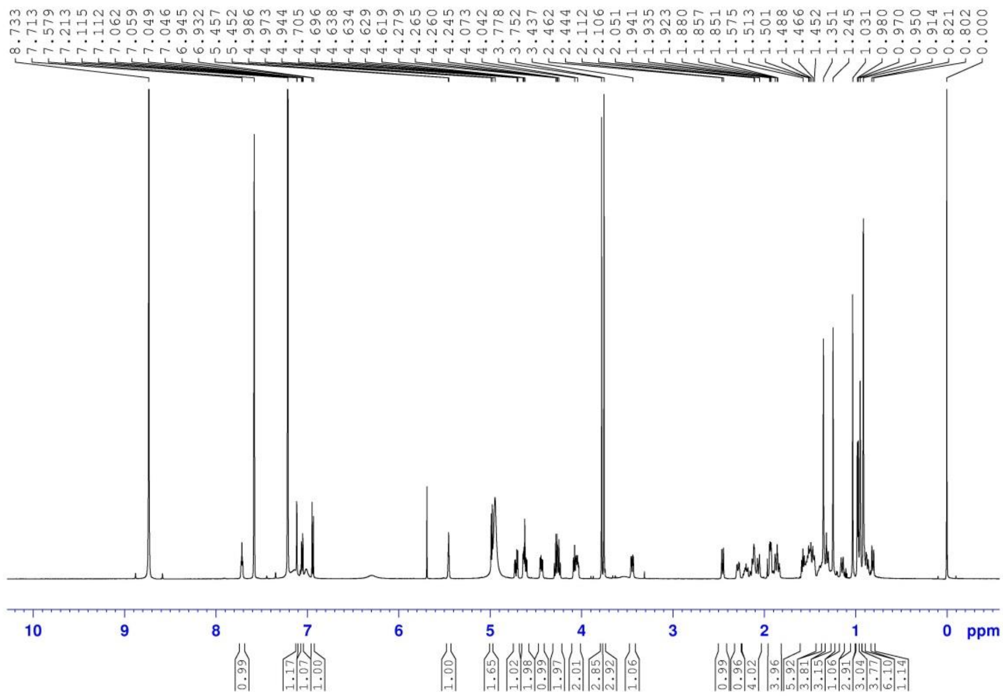

Figure S17 ^{1}H NMR of compound **7c**

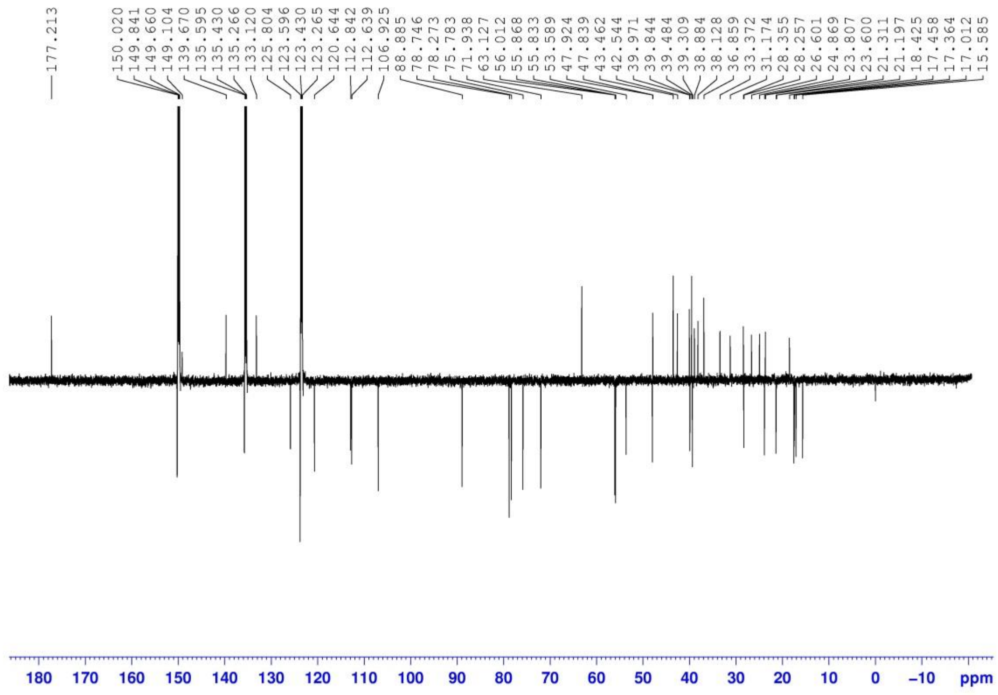

Figure S18 ^{13}C NMR of compound **7c**

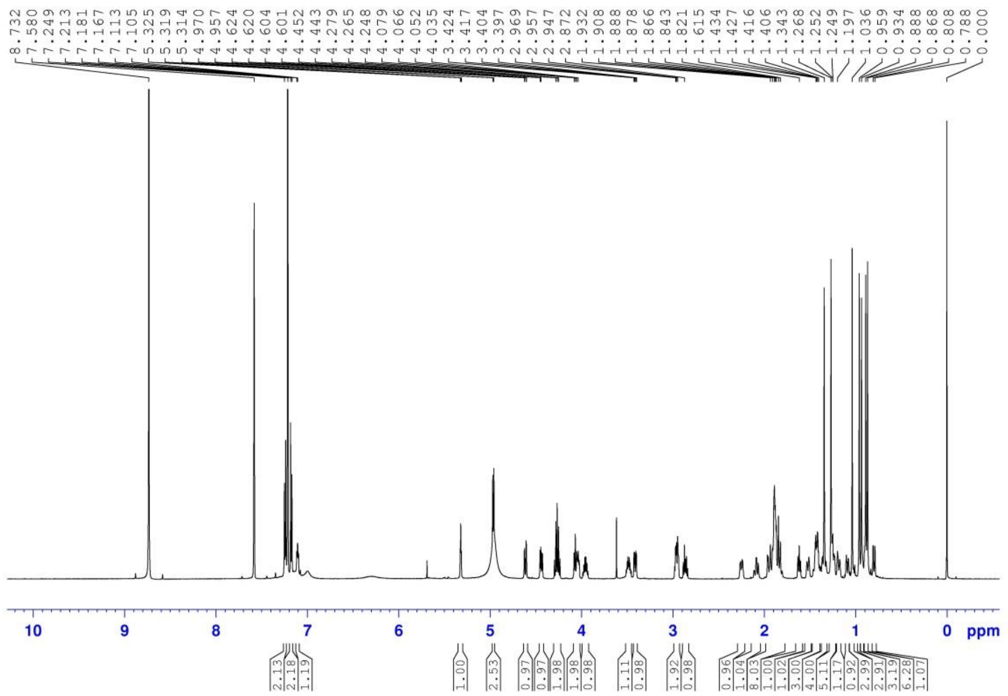

Figure S19 ^{1}H NMR of compound **8a**

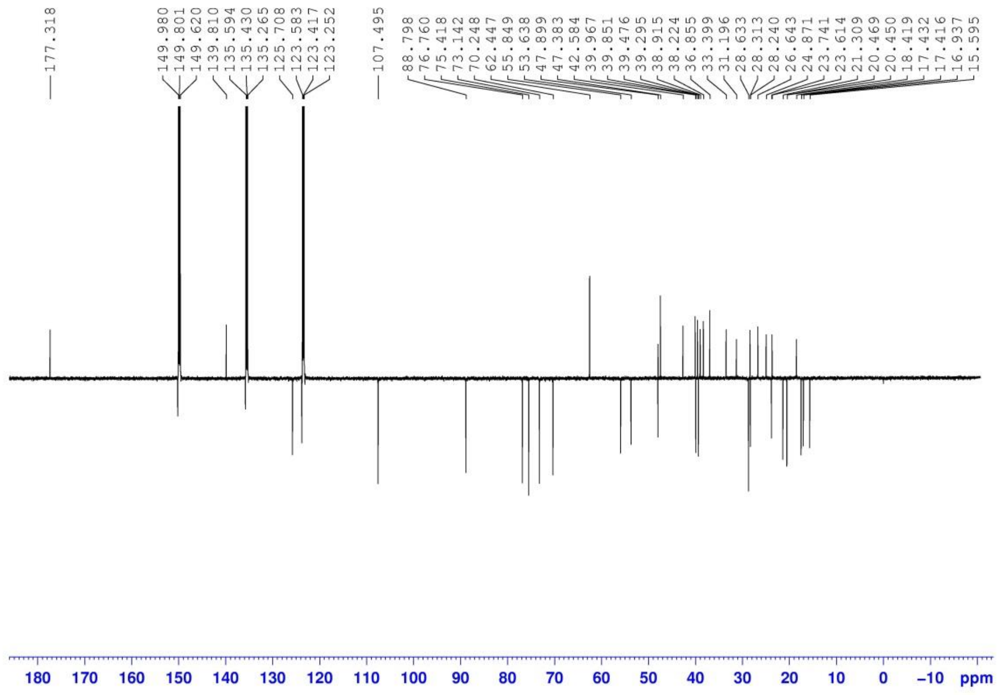

Figure S20 ^{13}C NMR of compound **8a**

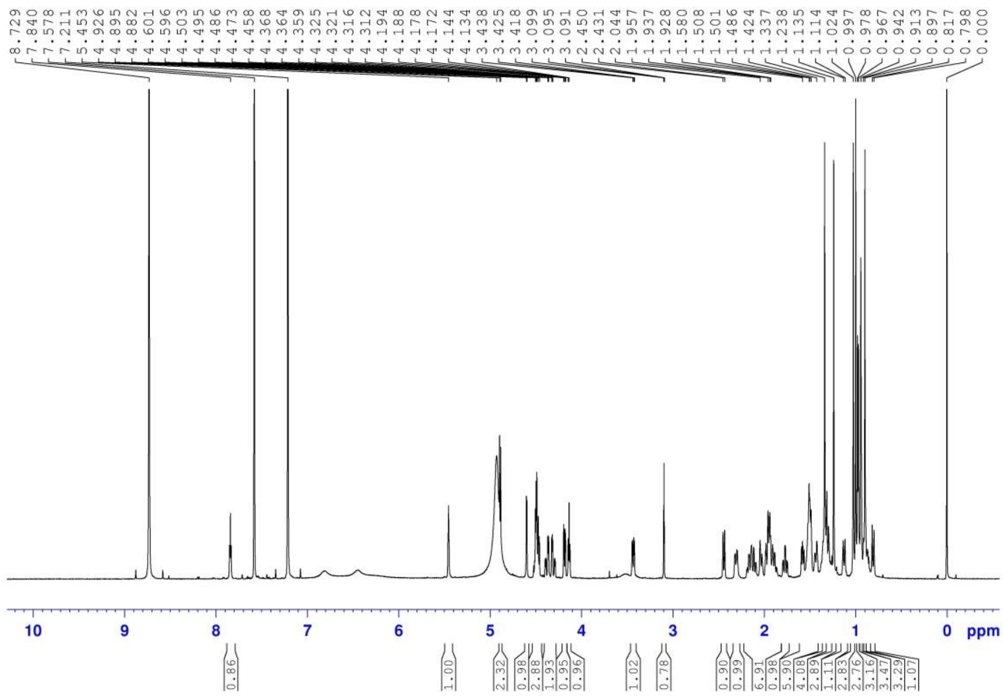

Figure S21 ^{1}H NMR of compound **8b**

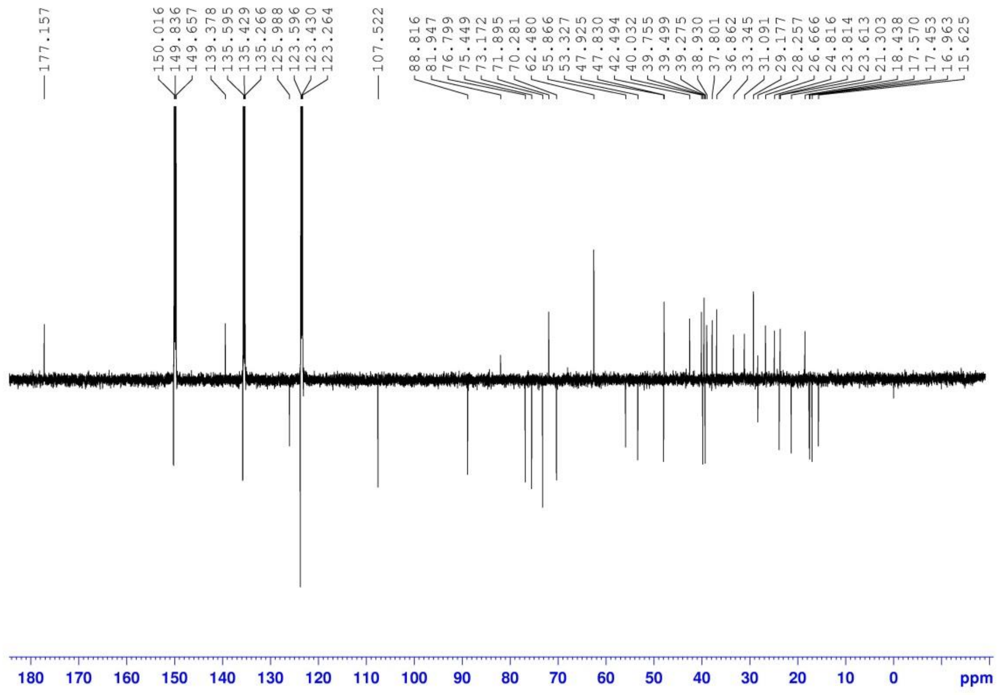

Figure S22 ^{13}C NMR of compound **8b**

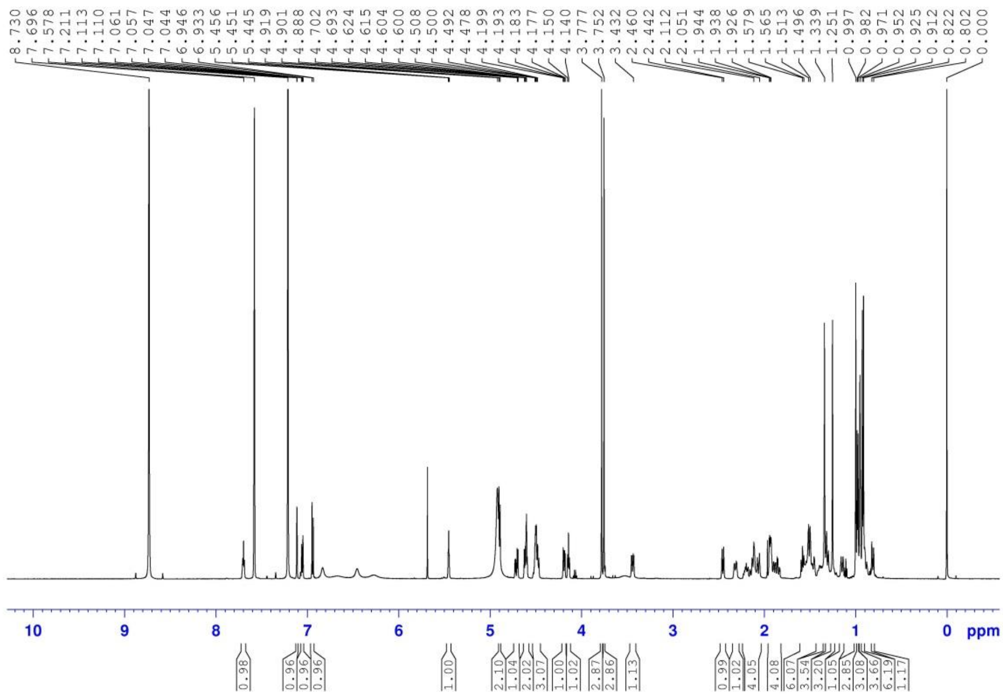

Figure S23 ^{1}H NMR of compound **8c**

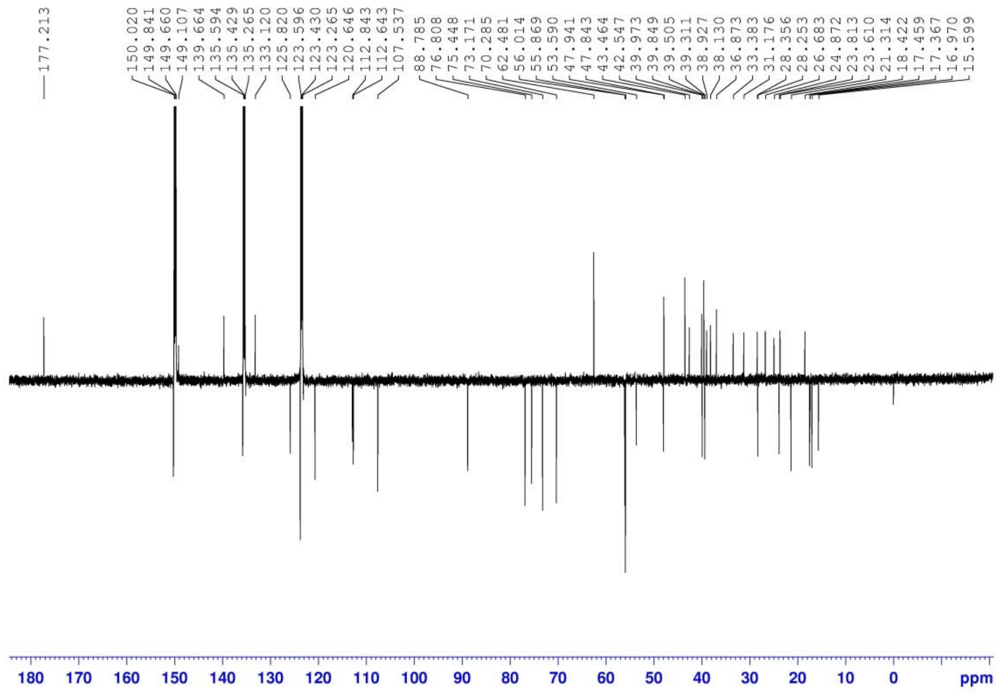

Figure S24 ^{13}C NMR of compound **8c**
